# Evolution and Taxonomic Classification of *Alphapapillomavirus 7* Complete Genomes: HPV18, HPV39, HPV45, HPV59, HPV68 and HPV70

**DOI:** 10.1371/journal.pone.0072565

**Published:** 2013-08-16

**Authors:** Zigui Chen, Mark Schiffman, Rolando Herrero, Rob DeSalle, Kathryn Anastos, Michel Segondy, Vikrant V. Sahasrabuddhe, Patti E. Gravitt, Ann W. Hsing, Robert D. Burk

**Affiliations:** 1 Department of Pediatrics, Albert Einstein College of Medicine, Bronx, New York, United States of America; 2 Division of Cancer Epidemiology and Genetics, National Cancer Institute, Bethesda, Maryland, United States of America; 3 Proyecto Epidemiológico Guanacaste, Fundación INCIENSA, San José, Costa Rica; 4 Sackler Institute of Comparative Genomics, American Museum of Natural History, New York, New York, United States of America; 5 Department of Medicine, Albert Einstein College of Medicine and Montefiore Medical Center, Bronx, New York, United States of America; 6 Departments of Epidemiology & Population Health and Obstetrics, Gynecology & Woman’s Health, Albert Einstein College of Medicine, Bronx, New York, United States of America; 7 Department of Biology and Pathology, Montpellier University Hospital, Montpellier, France; 8 Institute for Global Health, Vanderbilt University, Nashville, Tennessee, United States of America; 9 Department of Epidemiology, Johns Hopkins Bloomberg School of Public Health, Baltimore, Maryland, United States of America; 10 Cancer Prevention Institute of California, Fremont, California, United States of America; 11 Department of Microbiology & Immunology, Albert Einstein College of Medicine, Bronx, New York, United States of America; National Institute of Health - National Cancer Institute, United States of America

## Abstract

**Background:**

The species *Alphapapillomavirus 7* (alpha-7) contains human papillomavirus genotypes that account for 15% of invasive cervical cancers and are disproportionately associated with adenocarcinoma of the cervix. Complete genome analyses enable identification and nomenclature of variant lineages and sublineages.

**Methods:**

The URR/E6 region was sequenced to screen for novel variants of HPV18, 39, 45, 59, 68, 70, 85 and 97 from 1147 cervical samples obtained from multiple geographic regions that had previously been shown to contain an alpha-7 HPV isolate. To study viral heterogeneity, the complete 8 kb genome of 128 isolates, including 109 sequenced for this analysis, were annotated and analyzed. Viral evolution was characterized by constructing phylogenic trees using maximum-likelihood and Bayesian algorithms. Global and pairwise alignments were used to calculate total and ORF/region nucleotide differences; lineages and sublineages were assigned using an alphanumeric system. The prototype genome was assigned to the A lineage or A1 sublineage.

**Results:**

The genomic diversity of alpha-7 HPV types ranged from 1.1% to 6.7% nucleotide sequence differences; the extent of genome-genome pairwise intratype heterogeneity was 1.1% for HPV39, 1.3% for HPV59, 1.5% for HPV45, 1.6% for HPV70, 2.1% for HPV18, and 6.7% for HPV68. ME180 (previously a subtype of HPV68) was designated as the representative genome for HPV68 sublineage C1. Each ORF/region differed in sequence diversity, from most variable to least variable: noncoding region 1 (NCR1) / noncoding region 2 (NCR2) > upstream regulatory region (URR) > E6 / E7 > E2 / L2 > E1 / L1.

**Conclusions:**

These data provide estimates of the maximum viral genomic heterogeneity of alpha-7 HPV type variants. The proposed taxonomic system facilitates the comparison of variants across epidemiological and molecular studies. Sequence diversity, geographic distribution and phylogenetic topology of this clinically important group of HPVs suggest an independent evolutionary history for each type.

## Introduction

Genital human papillomavirus (HPV) infections constitute one of the most common sexually transmitted infections; however, only a subset of these cause cervical cancer and its immediate precursor, cervical intraepithelial neoplasia 3 (CIN3) [1]. Cervix cancer is the third most common cancer among women worldwide and is of particular importance in developing countries and based on the fact that relatively young women are stricken [2,3]. Most oncogenic or high-risk (HR) HPV types associated with invasive cervical cancer are phylogenetically clustered within the species groups *Alphapapillomavirus 9* (HPV16-related) or *Alphapapillomavirus 7* (HPV18-related) [4,5]. These two species groups account for approximately 75% and 15% of all cervical cancers worldwide, respectively [6–8].

The International Committee on Taxonomy of Viruses (ICTV) designates the definitions for genera and species groups of isolates within the family *Papillomaviridae* based on recommendations of an international PV working group committee. However, it does not set standards below the species level [9]. Within the PV research community, a distinct papillomavirus genotype is established when the nucleotide sequence of the L1 ORF of a cloned virus differs from that of any other characterized types by at least 10% [4,5]. However, the complete viral genome sequence is used to classify closely related HPV variants, since changes are not uniform across the genome. We have previously employed this nomenclature system to classify HPV6/11, HPV16 and HPV16 related alpha-9 HPV variants [10–12]; isolates of the same HPV type are grouped into variant lineages or sublineages when the pairwise nucleotide sequences of their genomes differ by 1.0%-10.0% or 0.5%-1.0%, respectively [12]. The lexicon of other taxonomic terms, such as serotypes, strains, subtypes, is not recommended in order to maintain a common language [4].

HPV variants have been shown to differ in geographic origins [13–16], evolutionary dynamics [17–21], and pathologic potential [15,16,22–24]. A comprehensive classification system can facilitate investigation of the clinical and biological role sequence variations play in genotype-phenotype associations. Moreover, since groups of single nucleotide polymorphisms (SNPs) are highly correlated within each lineage/sublineage, this allows HPV researchers to discuss the properties of HPV variant lineages without having to describe sets of nucleotide changes facilitating comparisons of data across studies. For example, an HPV33 variant (C7732G) and an HPV58 variant (C632T and G760A) reported to be associated with higher risks of cervical cancer [25,26] can now be classified into HPV33 sublineage A2 and HPV58 sublineage A3, respectively [12]. In addition, recent reports using the alpha-9 HPV variant nomenclature examined risk of cervical intraepithelial neoplasia grades 2-3 (CIN2/3) by HPV31 variants [27], which could be compared with other studies supporting the notion that the HPV31 C lineage was a risk factor for CIN2/3 [28].

In this report, the complete 8 kb genomes of 128 variants representing major lineages and sublineages of alpha-7 HPV types (HPV18, 39, 45, 59, 68 and 70) were selected and analyzed to capture maximum viral heterogeneity. Variations across the genomes are identified and the phylogeny and nomenclature of alpha-7 variant lineages/sublineages are described. Genomic diversity, evolutionary dynamics and geographic clustering are discussed.

## Materials and Methods

### Ethics Statement

The studies providing cervical samples for this work have been IRB approved by each ethics committee and all samples received in the Burk lab were coded and did not have individual identifying information. In details, **Rwanda** - The Rwanda National Ethics Committee and the Institutional Review Board of Montefiore Medical Center, Bronx NY approved the study protocol and the consent process. **Burkina Faso** - The Yerelon Cohort Research Programme was approved by the Ethical Committee of the Centre Muraz, Bobo Dioulasso, the National Ethical Committee of the Ministry of Health, Burkina Faso, and the Ethics Committee of the London School of Hygiene and Tropical Medicine, Zambia - The study was approved by the Research Ethics Committee of the University of Zambia and the Institutional Review Board of the University of Alabama at Birmingham, Thailand - The study protocols were reviewed and approved by the committees on human subject research at Johns Hopkins Bloomberg School of Public Health, Baltimore, MD; Merck & Co., Inc., West Point, PA, each participating recruitment site, and the Institutional Review Board of the Thailand Ministry of Health (MOH), Thailand. **Taiwan** - The study was approved by the Institution Review Board of the National Taiwan University University College of Public Health. **Guanacaste, Costa Rica** - The study and informed consent forms were approved by Institutional Review Boards of Costa Rica and the U.S. National Cancer Institute.

### Clinical Specimens, Identification of Novel HPV Variants and Whole Genome Sequencing

DNA from cervicovaginal samples already determined to have alpha-7 types (HPV18, 39, 45, 59, 68 and 70) by previous testing were available from women participating in epidemiological studies worldwide, including - Costa Rica [29], Taiwan [30], Thailand [31,32], Rwanda [33], Burkina Faso [34] and Zambia [35]. The methods for sample collection and HPV typing are provided in the references from each study. The number of samples analyzed for each type is shown in Table 1. The HPV genomes within the DNA samples were classified by sequencing the URR and/or E6 regions from PCR products as described [12,27]. Briefly, we used type-specific primers to amplify a partial fragment of the URR region and/or the E6 ORF using a one-tube nested PCR method [36]. The E6 ORF was evaluated only for those specimens that did not yield data for the URR region. The PCR product sizes were confirmed by gel electrophoresis, purified using the QuickStep 2 PCR Purification kit (Edge BioSystems, Gaithersburg, MD) or QIAquick Gel Extraction kit (Qiagen, Valencia, CA) and submitted for Sanger sequencing of both strands at the Einstein Genomics Facility. The sequences for each type were separately aligned and preliminary phylogenetic trees were used to identify samples that likely contained divergent viral genomes (data not shown). Based on this analysis, we selected type-specific viral isolates for complete genome sequencing that (1) represented novel variant clades or (2) had 2 or more isolates that contained at least 2 unique sequence variations (e.g., single nucleotide polymorphisms (SNPs)) not present in other isolates within the URR/E6 regions.

**Table 1:** Summary of HPV isolates, genome sizes, variability and variant lineages.

HPV type	Isolates tested^a^	Genomes analyzed ^b^	Genome size (nucleotide)^c^	Mean GC content (%)	Number of CpG site ^d^	Variable nt positions ^e^	Number of aa codons ^f^	Variable aa positions ^g^	Variant lineage / sublineage
HPV18	380	46	7857 (7824, 7857)	40.2	230 (2.9%)	422 (5.4%)	2476	165 (6.7%)	A1-A5, B1-B3, C
HPV39	122	20	7912 (7833, 7885)	40.2	183 (2.3%)	186 (2.4%)	2428	61 (2.5%)	A1-A2, B
HPV45	217	24	7858 (7841, 7858)	39.7	181 (2.3%)	239 (3.0%)	2440	89 (3.6%)	A1-A3, B1-B2
HPV59	45	8	7898 (7896, 7898)	38.7	164 (2.1%)	169 (2.1%)	2441	66 (2.7%)	A1-A3, B
HPV68	88	21	7850 (7814, 7836)	39.9	247 (3.1%)	771 (9.8%)	2419	232 (9.6%)	A1-A2, B, C1-C2, D1-D2, E, F1-F2
HPV70	295	9	7922 (7905, 7922)	40.3	172 (2.2%)	192 (2.4%)	2413	67 (2.8%)	A, B

^a^Number of isolates characterized by sequencing the URR ± E6 region;

^b^Number of complete genomes analyzed (including sequenced for this report, the prototype and other complete genomes available in NCBI/GenBank), see Table S1 for complete list with accession numbers;

^c^Number of nucleotides within the reference genome based on one genome size for each HPV type calculated from the global sequence alignments (see Materials and Methods). Minimum and maximum lengths of sequenced genomes for each type are shown and differ from the presence of insertions and deletions (indels);

^d^Number of CpG sites is the cumulative number in each alignment;

^e^Total number and percentage of variable nucleotide positions based on a reference genome for each HPV type as described above. Nucleotide variations include single nucleotide polymorphisms (SNPs) and indels, which are considered equivalent to one variation per indel, independent of indel size;

^f^Number of amino acid codons (not including overlapping ORFs) based on the reference genome for each type as described above. Cumulative number of amino acid codons are taken from 7 ORFs (E6, E7, E1, E2, E5, L2 and L1), E4 is not counted separately nor are other overlapping ORFs;

^g^Total number and percentage of variable amino acids based on the total number of amino acid positions derived from the reference genome for each HPV type.

The number of genomes selected for sequencing was based on identification of divergent isolates as described above and differed for each type (Table 1). The complete 8 kb genomes from clinical samples were amplified in 2 to 3 overlapping fragments using type-specific primer sets (available from authors) based on the prototype sequence of each type. For overlapping PCR, an equal mixture of AmpliTaq Gold DNA polymerase (Applied Biosystems, Carlsbad, CA) and Platinum Taq DNA Polymerase (Invitrogen, Carlsbad, CA) was utilized as previously described [12,37]. PCR products of anticipated size, as determined by gel analyses, were either directly sequenced or cloned into pGEM-T easy (Promega, Madison, WI) or TOPO TA pCR2.1 vectors (Invitrogen, Carlsbad, CA) and then sequenced. Comparison of repeat sequencing of PCR products from the same isolates resulted in a difference of less than one change per 8,000 bp; whereas, comparison of the cloned genomes gave a difference of approximately one difference per 5,000 bp. For discrepancies between sequences, we used the sequence of the PCR product as the consensus sequence. HPV complete genome sequences were submitted to GenBank (Table S1). In addition, a GenBank search, at the time of initial analysis (November 2012), for alpha-7 complete genomes identified 9 HPV18 isolates from Thailand [38], 1 HPV59 and 2 HPV68 isolates from China [39,40] and the set of reference sequences (i.e., HPV18, HPV39, HPV45, HPV59, HPV68 and HPV70.) ME180, previously a subtype of HPV68, was included as a variant of HPV68. HPV85 and HPV97 genomes were not investigated in this study due to the limited diversity of currently reported genomes [18]. The accession numbers of all sequences used in this report are listed in Table S1.

### Evolutionary analyses and phylogenetic tree construction

The nucleotide sequences of the complete circular genomes were linearized at the first ATG of the E1 ORF and globally aligned using the program MAFFT v 6.864b [41]. Based on the concept of a single ancestor for each type, a unique genome size is assigned to each HPV type based on the global alignment; the variation in genome sizes of isolated variants is the result of insertions and deletions (indels). Each indel was counted as one event. The assignment of position numbers for each nucleotide is based on the nucleotide numbering of the prototype reference sequence.

Maximum likelihood (ML) trees were constructed using RAxML MPI v 7.2.8.27 [42] and PhyML MPI v 1.4.3 [43] with optimized parameters based on the aligned complete genome nucleotide sequences. MrBayes v 3.1.2 [44] with 10,000,000 cycles for the Markov chain Monte Carlo (MCMC) algorithm was used to generate Bayesian trees. A 10% discarded burn-in was set to eliminate iterations at the beginning of the MCMC run. For Bayesian tree construction, the computer program ModelTest v 3.7 [45] was used to identify the best evolutionary model; the identified GTR model was set for among-site rate variation and allowed substitution rates of aligned sequences to be different. Data were bootstrap resampled 1,000 times in PhyML.

SNPs within the HPV genomes and lineage-specific SNPs were determined from alignments of type specific variant genomes using MEGA v 5.05 [46] and MacClade v 4.08 [47], respectively. Mean nucleotide differences and standard errors between and within type-specific lineages and sublineages were calculated from the global sequence alignment of each type using MEGA v 5.05 bootstrapped 1,000 times [46]. The p-distance method in MEGA v 5.05 [46] was used to calculate the genome-genome pairwise differences from the above alignments. The rarefaction curves for each type were generated by EstimateS v 8.2 [48].

## Results

### HPV variant distribution, lineage classification and nomenclature

**Figure 1:**
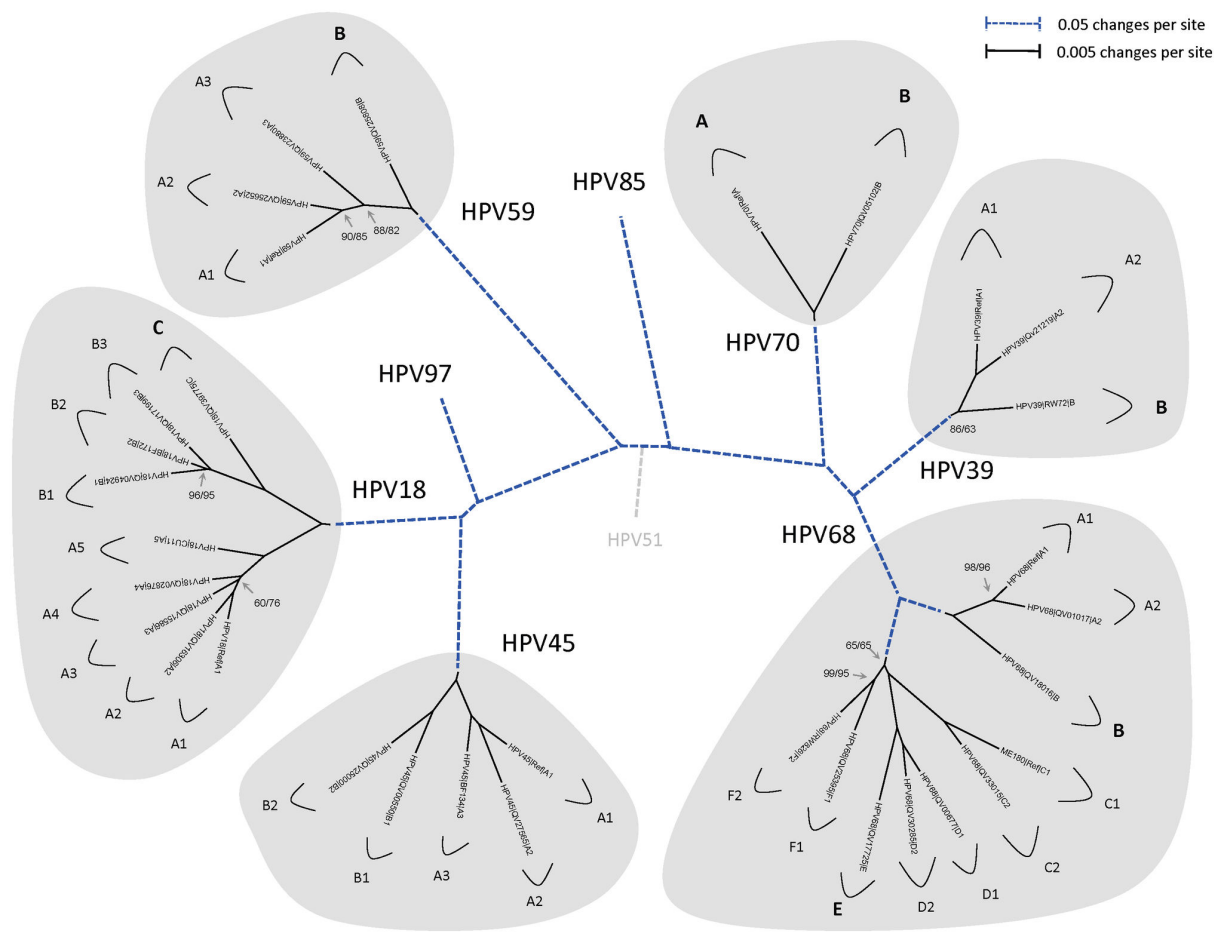
Alpha-7 phylogenetic tree showing representative types and variant lineages. A maximum likelihood (ML) tree was constructed using RAxML MPI v 7.2.8.27 [42] and PhyML MPI v 1.4.3 [43] inferred from the global alignment of complete circular genome nucleotide sequences linearized at the first ATG of the E1 ORF. Numbers on or near branches indicate support indices < 100% by RAxML and PhyML, respectively. The shaded areas represent groupings of lineages and sublineages of HPV18, HPV39, HPV45, HPV59, HPV68 and HPV70; the prototype sequences of HPV85 and HPV97 are also included as indicated, although no variant lineages are distinguished due to the limited number of isolates of these types. The length of broken and solid lines represent distance between clades, although the number of changes is different for these two lines, as indicated in the upper left corner of the figure. HPV51, an alpha-5 type, was set as the outgroup.

HPV isolates for sequencing were selected from a large set of samples previously typed for HPV and further analyzed for novel variants by sequencing a fragment of the URR and/or E6 ORF (Table 1). From the complete genome sequences of the isolates sequenced in this study and those obtained from GenBank, HPV variant lineage classification and nomenclature were assigned, as previously described [10,12]. The phylogenetic topologies for each type combined with an approximate cut off of 1.0% difference between complete genomes were used to define major variant lineages (1.0%-10.0%). Each major lineage was named using an alphanumeric, with the “A” clade always containing the reference genome for each type. Differences between genomes in the 0.5%-1.0% range were designated as sublineages (e.g., A1, A2, etc.). When the reference genome could be classified as a sublineage, it was always assigned into the “A1” clade. An overview of the alpha-7 HPV types and variant lineages are shown in Figure 1.

### Genomic Diversity of HPV18 Variants

**Figure 2:**
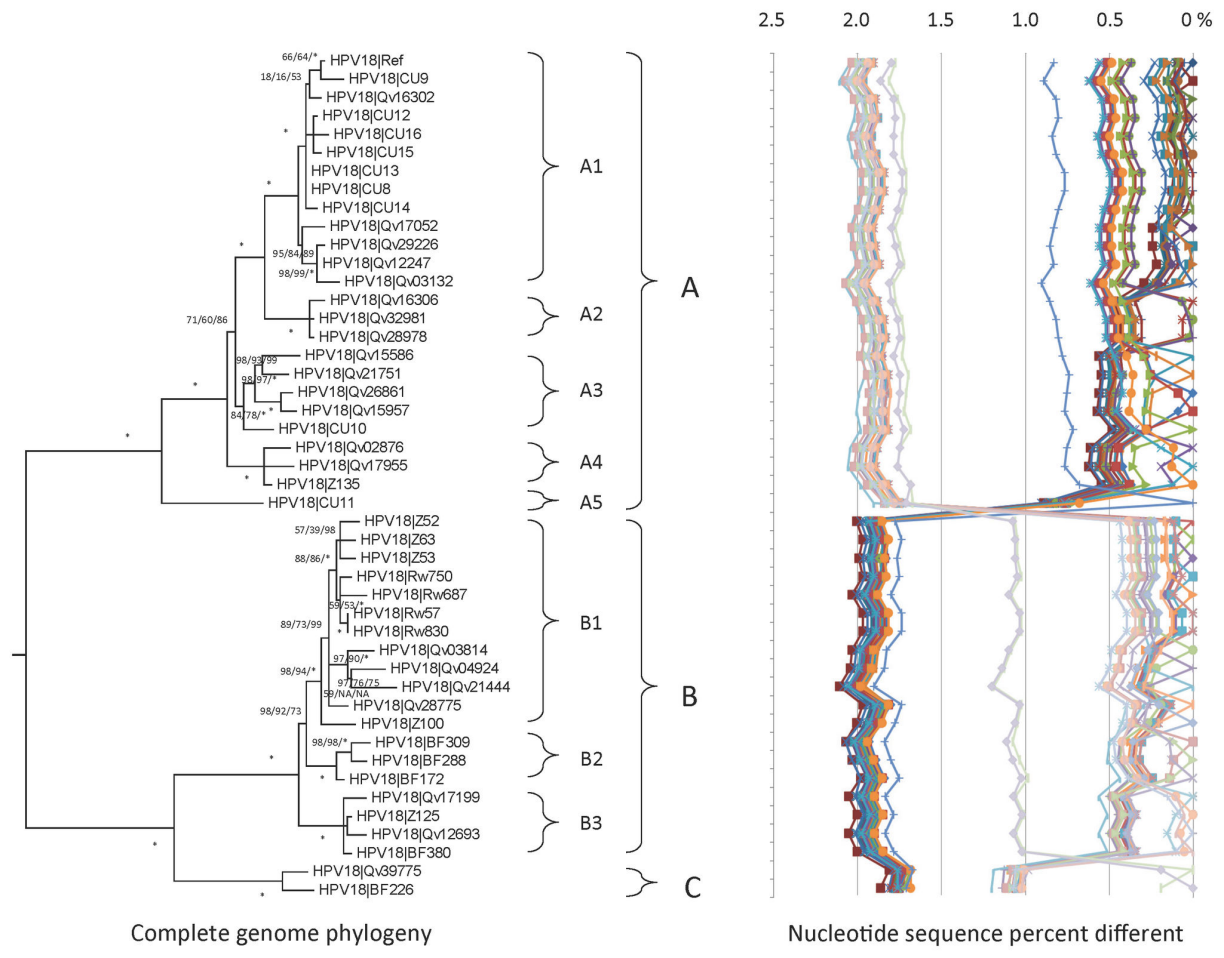
HPV18 tree topology and pairwise comparisons of individual complete genomes. Phylogenetic trees were inferred from global alignment of complete genome nucleotide sequences (the other alpha-7 HPV reference prototypes were set as the outgroup). Numbers on or near branches indicate support indices in the following order: maximum likelihood (ML) bootstrap percentages using RAxML MPI v 7.2.8.27 [42] and PhyML MPI v 1.4.3 [43], and Bayesian credibility value percentage using MrBayes v 3.1.2 [44]. An asterisk (*) indicates 100% agreement between methods. “NA” indicates disagreement between a method and the reference RAxML tree at a given node. Thus, one tree is shown, but three different methods of tree construction were used to estimate the support of the provided tree, as explained above. Distinct variant lineages (i.e., termed A, B, and C) are classified according to the topology and nucleotide sequence differences from > 1% to < 10%; distinct sublineages (e.g., termed A1 and A2) were also inferred from the tree topology and nucleotide sequence differences in the > 0.5% to < 1% range. The percent nucleotide differences for each isolate compared to all other isolates (i.e., 1 x 1 comparisons) are shown in the panel to the right of the phylogeny. Values for each comparison of a given isolate are connected by lines and the comparison to self is indicated by the 0% difference point. Symbols and colored lines are used to distinguish each isolate.

HPV18 complete genome variants have been previously investigated and two major lineages, European (E) and African (Af) were identified [14,18]. The URR and/or E6 regions of 380 HPV18 isolates from cervical samples were sequenced and clustered into distinct groups as shown in Figure 2 [14,18]. Numerous novel SNPs or SNP patterns, e.g., G437A, C7549A and T7475G (Figure S1A), were detected in several samples from which 23 variants with maximum diversity were selected for complete genome sequencing (Table 1). In addition, twenty-two HPV18 complete genome variants from Costa Rican (n = 13) [18] and Thai women (n = 9) [38] that were previously published (Table S1) are included in the current analyses.

A total of 422/7857 (5.4%) nucleotide positions showed variations whereas, 165/2476 (6.7%) codons specified variable amino acids (i.e., they represent nonsynonymous SNPs) (Table 1). The maximum nucleotide pairwise difference between the most dissimilar isolates was 2.1% (Table S2, second column). The most variable region was the noncoding region 2 (NCR2) between the E5 and L2 ORFs with 11.6% overall nucleotide diversity. Consistent with a previous report [18], three different indel events were detected across the genome in the following regions: the E2/E4 ORFs (6 bp deletion), the noncoding region 1 (NCR1 is the region between the stop codon of the E2 ORF and the start codon of the E5 ORF) (19 to 20 bp deletion), and the URR (7 bp deletion) (Figure S1A).

Phylogenetic analyses clustered HPV18 variants into two deeply separated groups (Figure 2). The previous non-Af variants were designated lineage A, and the majority of Af variants were classified as lineage B. A third new lineage was identified based on 2 isolates, Qv39775 and BF226; the complete genome nucleotide sequence differences of lineage C compared to lineage B variants were 1.0%-1.2% (Table S3). Lineage A was relatively distant to lineages B and C with mean differences of 1.92% ± 0.15% and 1.71% ± 0.14%, respectively. Lineage C variants could be distinguished by 23 SNPs throughout the complete genome (E6 - G437A; E1 - T1526C, A1636G; E2 – G3757C, C3790T; E5 – T3942C, T3963G; L2- T4423G, G4486A, A4513T, A4726G, G5191A, C5225A, G5351A; L1 – A5638G, G5764A, A5880G, T5942G, A6224T, T6536C, A6644C, A6845C; and URR- T7475G), while lineages A and B had 62 and 33 lineage specific nucleotide variations, respectively (Figure S1A and Figure S2).

Lineage A variants were further divided into 5 sublineages A1 - A5 with mean differences ranging from 0.37% ± 0.06% to 0.83% ± 0.10% (Figure 2 and Table S3). Previously termed HPV18 Asian-American (E1(AA)) variants clustered into sublineages A1 and A2; sublineages A3 and A4 variants were composed of European (E2) variants [18]. An isolate CU11 from a Thai woman [38] represented sublineage A5 (0.73%-0.83% different to other lineage A variants). The HPV18 prototype (HPV18|Ref) was assigned to the A1 sublineage. Lineage B could be subdivided into three sublineages: B1, B2 and B3 (Figure 2) based on tree topologies; sublineages B1/B2 and B3 have also been referred to as African-1 (Af1) and Af2, respectively [14,18].

The distribution of HPV18 variant lineages was consistent with previous geographic placement [14], however further classification of lineages revealed a few insights. Essentially all the isolates (96%, 24/25) from Taiwan and Thailand mapped to the A1 sublineage. Most isolates from southern-central African countries of Rwanda (62%, 16/26) and Zambia (50%, 6/12) were from the B1 lineage; whereas samples from Burkina Faso in western Africa were classified as B2 (46%, 5/11) or B3 (36%, 4/11). In contrast, the samples from Costa Rica showed a mixed distribution consisting of 45% (134/298) lineage A3, 22% (65/298) lineage B1 and 17% (50/298) lineage A1.

### Genomic diversity of HPV39 variants

We amplified and sequenced the E6/URR region of 122 HPV39-containing samples and selected 19 isolates for genome sequencing (Table 1 and Table S1). The overall nucleotide and amino acid variable positions of the complete genomes (19 sequenced for this analysis plus the reference sequence makes a total of 20 complete genomes) were 2.4% (186 sites among 7912 nt) and 2.5% (61 sites among 2428 aa), respectively. The noncoding regions (NCR2 and URR) were more variable than the coding ORFs. HPV39 isolates Tw562 and BF182 were the most distantly related genomes with a nucleotide sequence pairwise difference of 1.1%; this distance represented the maximum inter-lineage diversity of HPV39 variants (Table S2).

**Figure 3:**
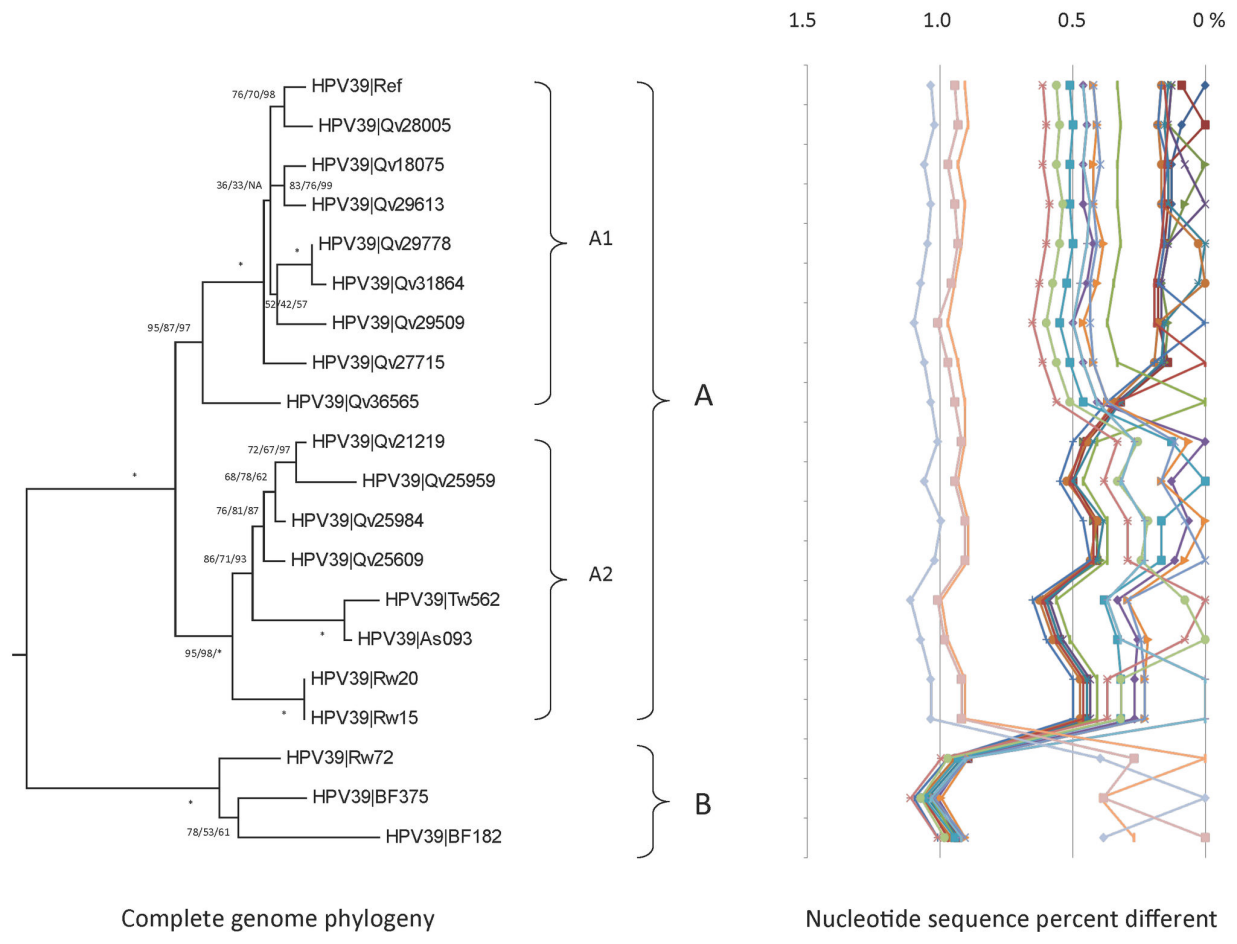
HPV39 variant tree topology and pairwise comparisons of individual complete genomes. The phylogenetic tree was constructed as described in Figure 2. Distinct variant lineages and sublineages were determined as described in Figure 2. The percent nucleotide sequence differences are shown in the panel to the right of the phylogenetic tree as described in Figure 2.

Phylogenetic analyses based on complete genome sequences clustered HPV39 variants into 2 lineages designated A and B (Figure 3). Lineage A was further divided into two sublineages, A1 and A2 (0.48 ± 0.06% different); both sublineages were nearly equally distant to lineage B, with mean differences of 0.98 ± 0.09% and 0.97 ± 0.08%, respectively (Figure 3 and Table S3). Isolate Qv36565 (an A1 variant) may bridge the evolution of sublineages A1 and A2, sharing a set of nucleotide changes with A2 variants (E1 - C1298G, C1683T, A2257C, G2355A; E2 – T3157C; E5 – A4081G; L1 – C6785A) (Figure S1B). Lineage and sublineage specific nucleotide variations were determine across the genomes (3 SNPs for sublineage A1, 7 for A2, 37 for lineage A, and 35 for lineage B) (Figure S2). Sublineage A2 variants had a nine amino acid repeat within the E1 ORF (DAEGE(H/N) GGS), and a 52 bp repeat was detected within the URR region of isolate Qv29509 (an A1 variant).

All HPV39 isolates from Costa Rica mapped to either the A1 (67%, 70/105) or A2 (33%, 35/105) lineages; whereas, the few samples from Taiwan sorted to A2 (2/4) or B (2/4), the Rwandan samples sorted to lineages A (7/8) or B (1/8) but all 5 samples from Burkina Faso mapped to lineage B.

### Genomic diversity of HPV45 variants

**Figure 4:**
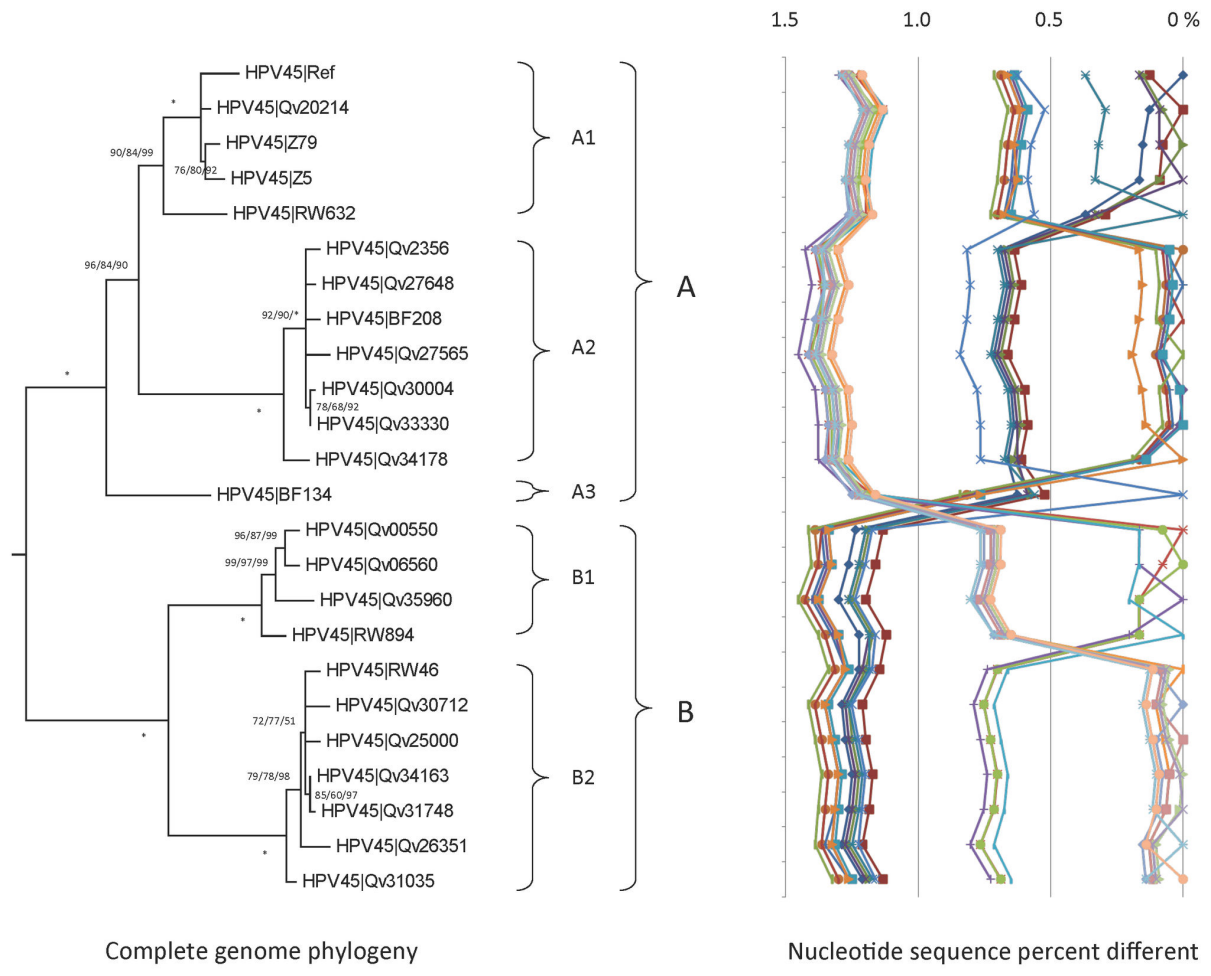
HPV45 variant tree topology and pairwise comparisons of individual complete genomes. The phylogenetic tree was constructed as described in Figure 2. Distinct variant lineages and sublineages were determined as described in Figure 2. The percent nucleotide sequence differences are shown in the panel to the right of the phylogenetic tree as described in Figure 2.

Eleven variants from 217 HPV45-containing samples were selected for complete genome sequencing; they represented 4 women from Costa Rica, 3 from Rwanda, 2 from Zambia, and 2 from Burkina Faso. Classification of HPV45 variants was based on the analysis of these samples combined with 13 previously reported HPV45 genomes, including the reference isolate [18,49]. There were a total of 239 nucleotide sites amongst the 7858 bp HPV45 genome that were variable (3.0%). Of the 2440 encoded amino acids, 89 (3.6%) showed variations (Tables 1 and S2, Figure S1C). The maximum nucleotide difference was 1.5%; the most divergent isolates were Qv27565 (an A2 variant) and Qv35960 (a B1 variant); this distance represented the maximum diversity between lineage A and B variants (Table S2 and Figure 4).

Isolate BF134 (sublineage A3) from a Burkina Faso woman had unique variations and was 0.57%-0.80% and 1.19% -1.21% different than the characterized A1/A2 and B1/B2 variants, respectively (Table S3). Based on the phylogenetic tree topology, BF134 separated from the precursor of the A1/A2 sublineages (Figure 4). Lineage A and B variants are distinguished by 42 SNPs across the genome (Figure S1C). In addition, several sets of SNPs are diagnostic for sublineages A1 (n = 3), A2 (n = 20), A3 (n = 20), B1 (n = 17), and B2 (n = 17) using the current 24 genomes (Figure S2).

HPV45 isolates from Costa Rica mapped to the A2 (37%, 64/171) or B1 (40%, 68/171) sublineages, samples from Thailand sorted to the B1 (62%, 5/8) or B2 (38%, 3/8) sublineages, samples from Rwanda were predominantly B2 (80%, 8/10), whereas samples from Zambia were predominantly A1 (45%, 10/22) and B2 (36%, 8/22) and those from Burkina Faso A1 (67%, 4/6).

### Genomic diversity of HPV59 variants

**Figure 5:**
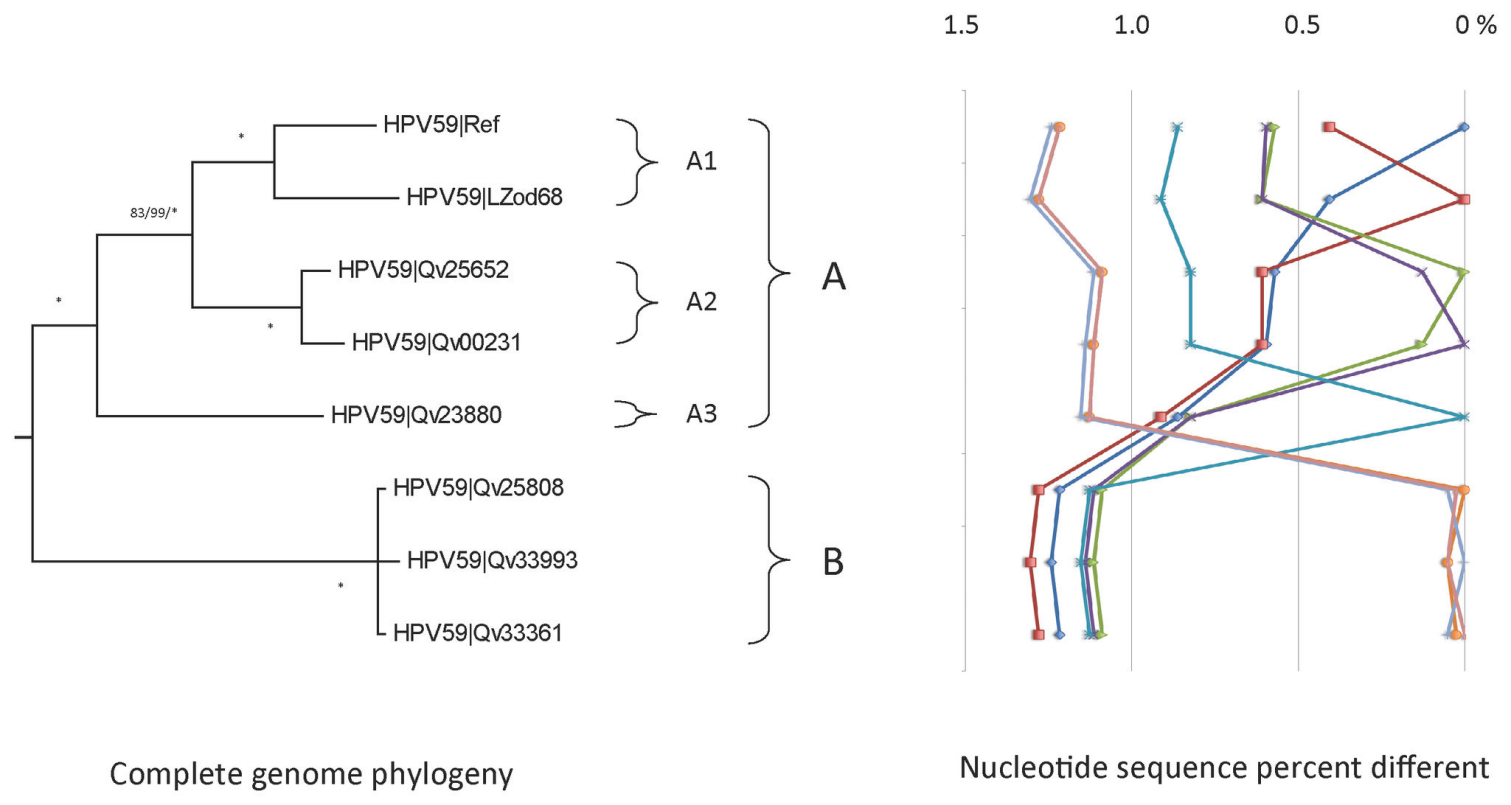
HPV59 variant tree topology and pairwise comparisons of individual complete genomes. The phylogenetic tree was constructed as described in Figure 2. Distinct variant lineages and sublineages were determined as described in Figure 2. The percent nucleotide sequence differences are shown in the panel to the right of the phylogenetic tree as described in Figure 2.

Forty-five HPV59 isolates had the URR/E6 regions sequenced; six samples encompassing each unique variation pattern were selected for complete genome sequencing (Table 1). In total, 8 complete genomes, including the prototype [50] and one isolate from a Chinese woman [40] were analyzed. There were 169/7898 (2.1%) variable nucleotide positions and 66/2441 (2.7%) variable amino acids (Table S2 and Figure S1D). The maximum pairwise nucleotide difference was 1.3%, which was observed between isolates LZod68 and Qv33993 (Table S2); these divergent isolates were part of HPV59 sublineage A1 and lineage B, respectively, as inferred from the phylogenetic analysis (Figure 5). Isolates within lineage A were more variable than those from lineage B and were further classified into sublineages A1, A2 and A3, with mean differences of 0.60%-0.89% (Table S3). HPV59 lineages A and B had mean differences of 1.11%-1.26% and can be distinguished by 53 lineage-specific SNPs across the genome (Figure S2).

Inspection of the distribution of the forty-five HPV59 variants from 4 geographic regions indicated that 61% (20/33) of variants from Costa Rica were A2, whereas 67% (4/6) and 60% (3/5) of samples from Rwanda and Burkina Faso were of the B lineage, respectively (data not shown). Thus, the B lineage was more common in samples from Africa, but the numbers were limited and the difference was not statistically significant (p = 0.11).

### Genomic diversity of HPV68 variants

**Figure 6:**
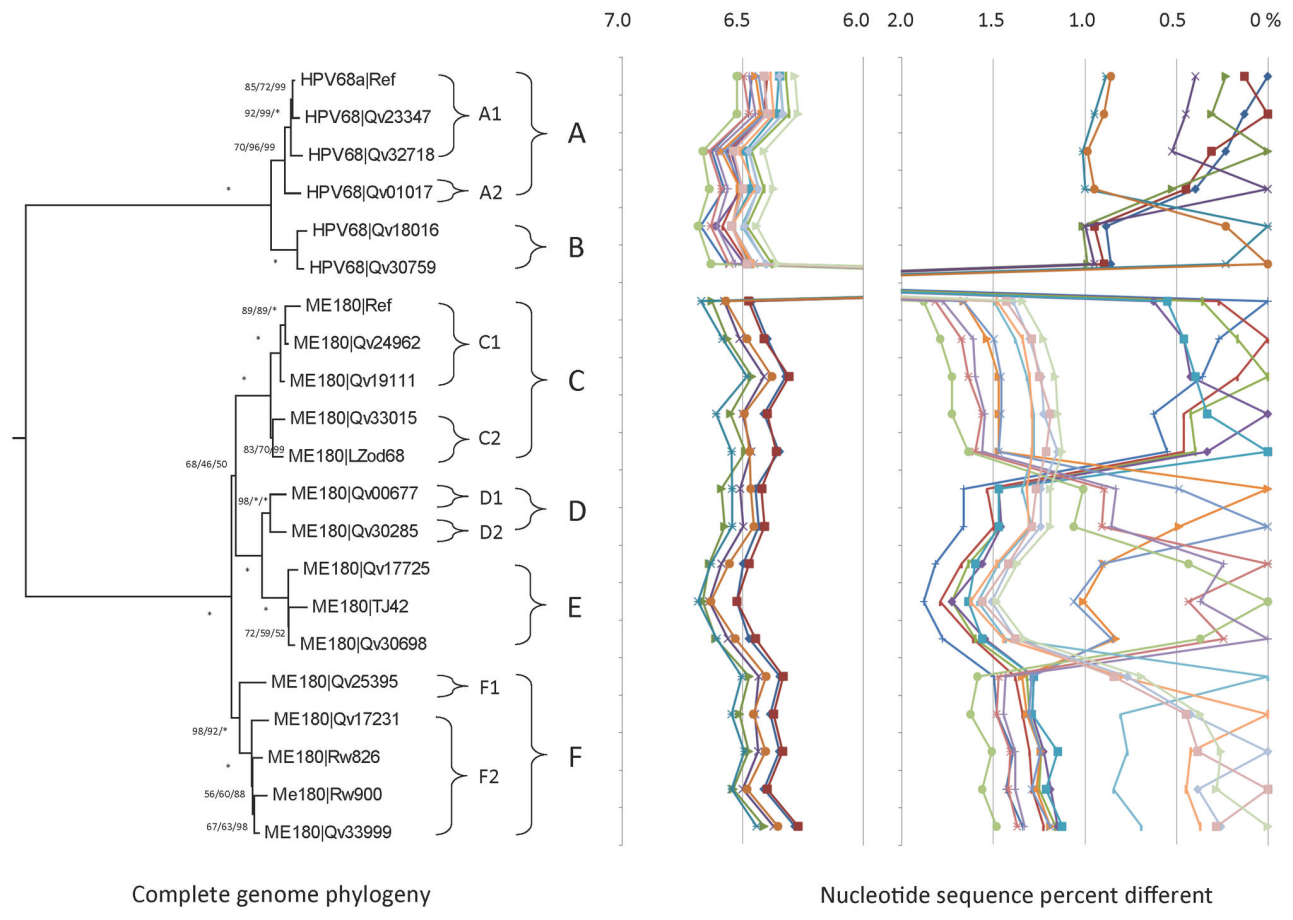
HPV68 variant tree topology and pairwise comparisons of individual complete genomes. The phylogenetic tree was constructed as described in Figure 2. Distinct variant lineages and sublineages were determined as described in Figure 2. The percent nucleotide sequence differences are shown in the panel to the right of the phylogenetic tree as described in Figure 2.

Historically two isolates of HPV68 (HPV68Ref(R)/HPV68a and ME180/HPV68b) were characterized, shared 93% nucleotide sequence similarity within the L1 ORF, and were designated as subtypes [51,52]. We sequenced the URR and/or E6 regions of eighty-eight HPV68 positive samples from patients in Costa Rica, Taiwan, Rwanda and Burkina Faso, and identified 20 isolates (22.7%) related to HPV68R, and 68 (77.3%) related to ME180. All isolates (5 HPV68R-related and 12 ME180-related) representing unique variation patterns were sequenced. Additionally, four HPV68 complete genomes from NCBI/GenBank, including the 2 prototypes [51,52], are included in this report (Figure 6, Table 1 and Table S1).

The maximum pairwise difference, 6.7% across the complete genome and 7.8% within the L1 ORF, was between isolates from lineage B, Qv18016 (an HV68a variant) and lineage E, TJ42 (a ME180 variant) (Table S2). Overall, 9.8% (771/7850) of nucleotide sites and 9.6% (232/2419) of encoded amino acids were variable (Table S2, Figure S1E). Based on lineage assignment encompassing 1.0%-10.0% differences in complete genomes [12], the “subtype” designation was replaced by assigning variant lineages to this taxon. Hence, ME180-related genomes were assigned to HPV68 variant lineages C, D, E and F, and HPV68R-related isolates to lineages A and B.

HPV68 lineages A/B are distantly split from lineages C/D/E/F (Figure 6); all variants sorted into the two distinct groups and displayed mean differences of 6.38%-6.61% across the complete genomes (Table S2, Figure S2). Comparison of the intragenomic diversity of variants within clades A/B and C/D/E/F showed differences of 0.93%-0.97% and 1.20% to 1.72%, respectively (Table S2). Thus, the ME180-related variants were more variable than the HPV68R-related isolates, consistent with the phylogenetic analysis that two HPV68R-related lineages and four ME180-related lineages constitute HPV68 (Figure 6). The following sublineages further classify HPV68 isolates: A1 and A2, C1 and C2, D1 and D2, and F1 and F2 (Figure 6). The isolates from Burkina Faso mapped to lineages D2 (1/6), E (2/6) and F (3/6); the isolates from Rwanda mapped to A1 (2/14), B (1/14), C1 (7/14), E (1/14) and F (3/14); and all four isolates from Taiwan sorted to C2. Thus, 85% of isolates available from Africa were from the C/D/E/F clade, whereas 70% (47/67) of the isolates from Costa Rica were from the C/D/E/F clade (p = 0.19).

### Genomic diversity of HPV70 variants

Amplification and sequencing the URR and/or E6 regions of 295 samples containing HPV70 clustered isolates into two distinct groups from which 8 representative isolates were selected for complete genome analyses, in addition to the reference sequence [52] (Table 1). In total, 192 nucleotide variable positions were observed across the 7922 bp genome (2.4%) and 67 of 2413 (2.8%) encoded amino acids were variable (Table S2). The maximum pairwise nucleotide difference was 1.6% between Qv27211 and Qv17574 (Table S2). Within the HPV70 genomes, we detected indels within the NCR2 and URR regions, and a 6-bp insertion (ACTGTA) between nt 6123 and 6124 within the L1 ORF resulting in an insertion of threonine and valine, between L1 aa position 178 and 179 (Figure S1F).

**Figure 7:**
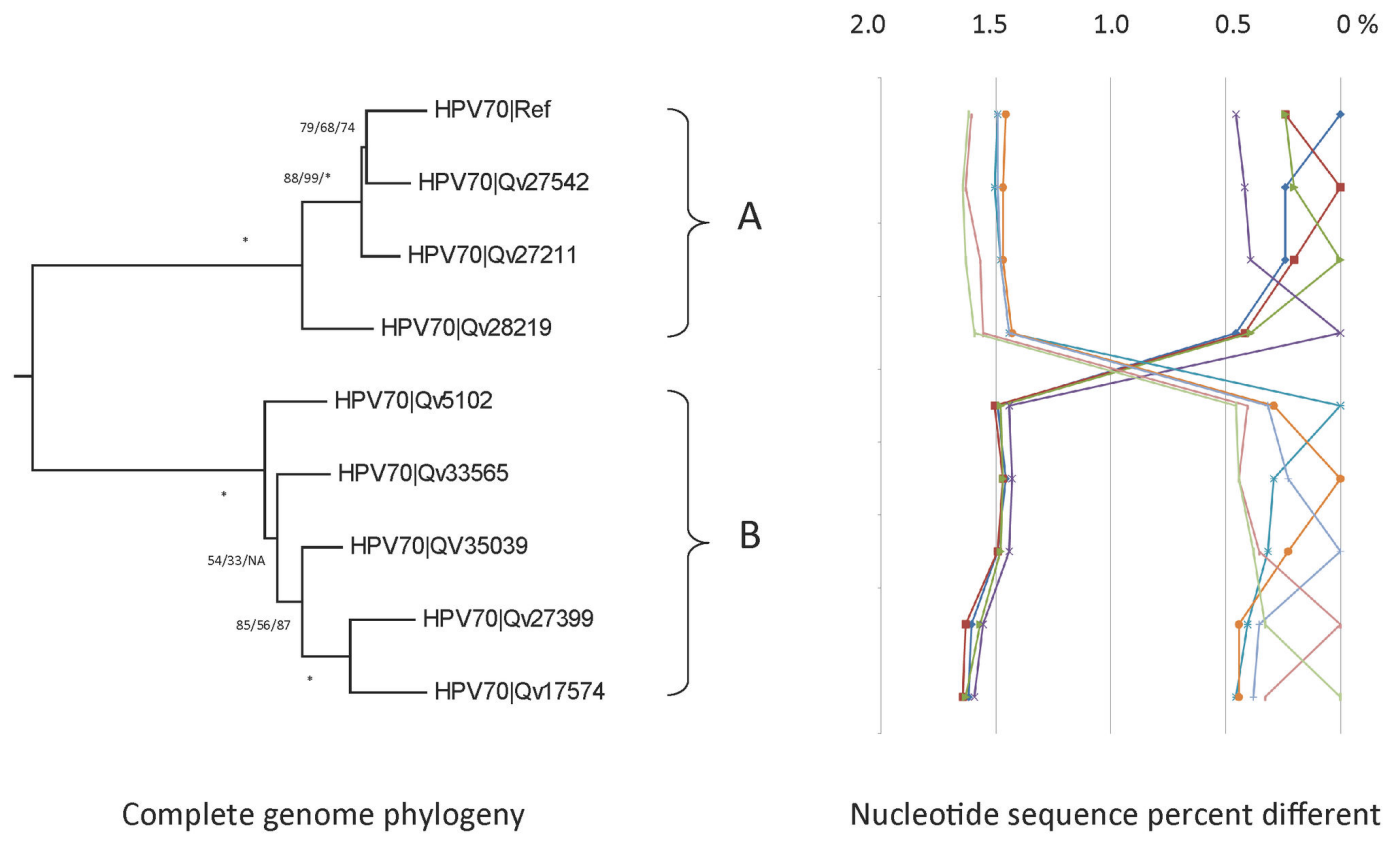
HPV70 variant tree topology and pairwise comparisons of individual complete genomes. The phylogenetic tree was constructed as described in Figure 2. Distinct variant lineages and sublineages were determined as described in Figure 2. The percent nucleotide sequence differences are shown in the panel to the right of the phylogenetic tree as described in Figure 2.

Phylogenetic trees inferred from HPV70 isolate nucleotide sequences separated variants into two distinct lineages, A and B (Figure 7). These lineages differ by 1.52 ± 0.13% and are distinguished by 82 lineage specific SNPs (Table S2, Figure S2). Isolates from the A lineage differed between geographic region: Rwanda 82% (18/22), Costa Rica 65% (175/268) and Burkina Faso 40% (2/5).

### Alpha-7 HPV evolution

**Figure 8:**
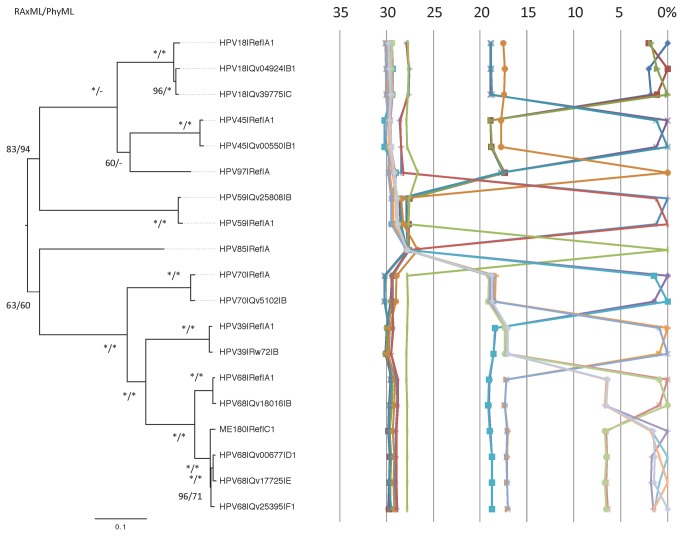
Phylogenetic tree and pairwise comparisons of representative Alpha-7 lineages and sublineage complete genomes. The phylogenetic tree was constructed as described in Figure 2. Representative genomes for each variant lineage and sublineage described in this report are used and the names of the isolates are shown to the right of the tree branches. The percent nucleotide sequence differences are shown in the panel to the right of the phylogenetic tree as described in Figure 2.

To investigate the relationship between isolates from the alpha-7 species group, we took one representative genome from each lineage or sublineage, constructed a phylogeny and plotted the percent genome differences from global alignments (Figure 8). Four clades were apparent from this analysis- HPV18/45/97, HPV59, HPV85 and HPV39/68/70 corresponding to the 4 deep-seated branches leading to these clades. Each clade was approximately 30% different from isolates within other clades, whereas the clades with more than one type (i.e., HPV18/45/97 and HPV39/68/70) consistently shared at least 80% of nucleotides. The intratype differences are displayed as a heatmap (Figure S3).

### Alpha-7 HPV genomic diversity

Rarefaction curves of parsim-informative SNPs (sites detected in ≥ 2 samples) for combined genomes of each type were plotted to estimate the coverage of type specific variations within the sampled cohorts and genomes analyzed (Figure S4A). Although sampling of genomes within the targeted populations may increase the repertoire of genomic variability, it is unlikely to reveal novel variant lineages since the curves flatten out with increasing numbers of genomes already sequenced. Interestingly, the detection of singleton SNPs (variations present only once in the sampled genomes) increases almost linearly with increasing sample size of each type (Figure S4B). Rare variations add information on mutations that potentially occurred during recent epochs of explosive population growth or natural selection and may be identified from sequencing larger sample sizes [53]. As sequencing larger numbers of isolates becomes more economically feasible with Next-Gen technologies, false positive SNPs within the genomes of additional variants remains unavoidable due to errors introduced by PCR amplification, sequencing technologies, software analyses and other unknown causes of DNA sequence error.

The pairwise inter-lineage mean differences inferred from the genome sequences revealed maximum genomic diversity of HPV68 and HPV18, followed by HPV70, HPV45, HPV59 and HPV39. When each ORF/region was compared, the noncoding regions (NCR1, NCR2 and URR) most often showed the largest variability, followed by the E5 and E4/E2 overlapping ORFs (Table S2). The diversity of the E6, E7, E1, E2, L2 and L1 ORFs varied by type; nevertheless, E1 and L1 ORFs were more conserved in terms of the overall nucleotide diversity compared to the other ORFs (Table S2). Indel events were observed within both coding and non-coding regions, resulting in variability in length of specific ORFs/regions among variants of the same types (Figure S1 and Figure S5).

## Discussion

Alpha-7 (HPV18, 39, 45, 59, 68, 70, 85 and 97) is the second most important HPV species group with regard to carcinogenicity. In an effort to investigate and understand the genetic basis of HPV pathogenicity, we have sought to establish the genetic heterogeneity of PVs derived from the common ancestor to all HPV-related oncogenic types [17,18,54–56]. To this end, we describe in this manuscript the characterization of a set of full viral genomes from the alpha-7 species either sequenced in our lab or obtained from GenBank. An overview of the tree topology (Figure 1) indicates that each type has a very different evolutionary history. This is further supported by the different diversities of isolates from each type (e.g., 1.1% for HPV39 and 6.7% for HPV68) and surprising varied geographic distribution of lineages and sublineages. Nevertheless, there are some common features that indicate residual punctuated evolution. These include 3 levels of heterogeneity, suggesting at least three evolutionary events including – (i) the rapid expansion of the host population resulting in viral variant lineages and sublineages predominantly in the < 3% range corresponding to events occurring approximately 0.2-1.0 million years ago (MYA) as previously reported [18]; (ii) a subspecies event accounting for the difference between types in the HPV18/45/97 and HPV39/68/70 clades of approximately 18% corresponding to events occurring approximately 5-10 MYA [18], and (iii) the speciation of the alpha-7 most recent common ancestor (MRCA) that resulted in the approximately 30% difference between members of the different alpha-7 clades corresponding to events occurring approximately 10-20 MYA [18]. Each alpha-PV species group has its own unique properties of evolutionary topology, diversity and geographic distribution of variant lineages. For comparison, the alpha-9 HPV clades differ by approximately 27%, whereas when all species groups within the alpha-HPV taxon are considered, they differ by approximately 40% ± 5%. However, since we have sampled from extant circulating alpha-7 HPVs and although we have tried to capture maximum viral heterogeneity, it is possible that additional lineages and sublineages will be discovered. Nevertheless, we do not believe that discovery of additional alpha-7 genomes will impact the basic observations derived from the currently available genomes.

This work reports the analysis of a large set of alpha-7 complete genomes from the additional sequencing of 109 variants from 1147 alpha-7 HPV isolates plus 19 complete genomes available from GenBank. These genomes were sampled from > 12,000 women in the Americas, Africa and Asia that were tested for HPV infection and subsequently selected based on the analysis of the URR/E6 regions to identify samples representing the major variant lineages and the maximum genomic diversity. We used a complete genome nucleotide sequence difference of approximately 1.0%-10.0% between two or more variants of the sample type to define distinct variant lineages. Similarly, differences across the complete genome of 0.5%-1.0% were used to distinguish sublineages. In contrast to genera, species and type definitions based on the L1 ORF nucleotide sequence, complete genome alignments are used to classify variant lineages, since the recently evolved variant genomes have changes that are not evenly distributed throughout the genome.

Similar to HPV16-related alpha-9 species variants, isolates of HPV18, 39, 45, 59, 68, 70 form at least two deeply separated clades implying codivergence of archaic Hominid and HPV variants. The maximum differences between lineages within a given type were variable (1.1%-6.7%); this most likely reflects different divergence times when isolates of a specific type split from their most recent common ancestor (MRCA). Alternatively, uncharacterized lineage or sublineage variants might exist in an isolated and/or unsampled population or could have disappeared by genetic isolation and/or host demise. Nevertheless, discrete events in human evolution are captured in the patterns of HPV variability.

The evolution and pathogenesis of HPV18 and HPV45 variants have been previously reported [14,18]. However, the epidemiological and pathological characteristics of other alpha-7 variant lineages warrant further study. Natural variation exists within all populations of organisms. This occurs partly because random mutations cause changes in the genome of an individual organism, a significant portion of genetic variation is functionally neutral in that it produces no phenotypic effect or significant difference in fitness, while some variations or variation patterns affect a phenotype and could be the result of niche adaptation or a function of fitness. Given the low mutation rate of papillomaviruses [57], natural selection remains an important mechanism for adaption of this slowly evolving virus. However, man-made selection could be a potential force driving the emergence of new SNPs or variant lineages with the introduction of VLP vaccines. Based on what we know about the evolution of the HPV genome, it is unlikely that HPV will show significant change in response to the vaccine, since there is no known mechanism for the virus to develop an increased rate of mutation as it uses the host replication machinery. Nevertheless, empirical data will be needed to assess the underlying evolution of HPV variants in the face of immune pressure generated by the vaccines; whether we will observe the emergence of new variants or variant lineages, or the extinction of particular lineages or types over time remains to be determined.

## Supporting Information

Table S1 **List, description and NCBI number of alpha-7 genomes.**
(PDF)

Table S2 **Variability of alpha-7 HPV genome regions and open reading frames (ORFs).**
(PDF)

Table S3 **Comparison of nucleotide sequence differences between variant lineages and sublineages for each type.** The intra-lineage (e.g., A vs. A) and intra-sublineage (e.g., A1 vs. A1) difference values are highlighted in gray.
(PDF)

Figure S1 **Sequence polymorphisms at nucleotide and amino acid positions within the complete genomes and ORFs of alpha-7 HPV variants.** Amino acids alignments were used to guide the nucleotide sequence alignments. The original GenBank sequence for each type is used as the reference for all alignments and is shown at the top of each panel. Only sites that are different from the reference sequence in one or more of the isolates (name is on the left of the panel with the type|sample identifier|lineage or sublineage listed) are displayed. Below the nucleotide sequence alignments are the corresponding amino acid differences for each ORF. The nucleotide sequence variations are shown for each position listed at the top of the panel by ORF or region. Under the reference sequence the nucleotide sequence of each isolate is displayed listing only sites that are different. Dots, sites matched with reference sequence; dashes, indel events. NCR1, noncoding region between E2 and E5 ORFs; NCR2, noncoding region between E5 and L2 ORFs; URR, upstream regulatory region located between stop codon of L1 and start codon of E6. Genome sequences for each lineage or sublineage are alternatively shown as grey blocks for visualization of most closely related isolates: (A) alignment of HPV18 complete genomes, (B) alignment of HPV39 complete genomes, (C) alignment of HPV45 complete genomes, (D) alignment of HPV59 complete genomes, (E) alignment of HPV68 complete genomes, and (F) alignment of HPV70 complete genomes.
(PDF)

Figure S2 **Diagnostic lineage- and sublineage-specific single nucleotide polymorphisms (SNPs) of alpha-7 types.** Lineage-specific SNPs were determined from alignments of type specific variants using the program MacClade. The location of variants across HPV lineage(s) and sublineage(s) are displayed to the right of the name of the clade from which the data were generated, as depicted in the phylogenetic trees in Figures 2-7. Numbered positions of SNPs in the genomes are shown in Figure S1. Regions of the genome are displayed below the x-axis for reference. The graphic output was generated using Microsoft Excel.
(PDF)

Figure S3 **Heatmap display of alpha-7 HPV variant genome nucleotide sequence differences.** P-differences calculated based on the complete genome nucleotide sequence alignment of each type were measured and represented as a heatmap, scaled such that complete identity (0.0% difference) is displayed as blue and the maximum difference (2.1%) as red. A phylogenetic tree indicating the position and name of each lineage and sublineage is shown above the heatmap.
(PDF)

Figure S4 **Rarefaction curves of alpha-7 HPV parsim-informative (A) and singleton (B) SNPs.** The program EstimateS was used to illustrate the curves. The Y-axis represents the total number of SNPs (indels were counted as one event equal to a single SNP). The X-axis shows the number of sequenced isolates. The curve generated for variants of each HPV type are displayed by different lines as indicated by the key to the right of the curves. HPV68 variants were split into two groups, HPV68A/B and HPV68C/D/E/F (HPV68-ME180). For reference, the number of variable nucleotide positions for HPV18, HPV39, HPV45, HPV59, HPV68 and HPV70 genomes are 5.4%, 2.4%, 3.0%, 2.1%, 9.8% (1.5% of HPV68A/B, and 4.2% of HPV68C/D/E/F) and 2.4%, respectively (Table 1).
(PDF)

Figure S5 **Representation of an alpha-7 HPV genome and type-specific ORF/region sizes.** Each region or ORF of the HPV genome is indicated outside the double-stranded circle. Lengths of each ORF and region are indicated by the histogram pointing to the region/ORF in the figure. The length in nucleotide sequences (bp) for each alpha-7 HPV genome is indicated with the minimal and maximal lengths represented by the bars with dots or highlighted in grey, respectively. The diagram of the HPV genome is not drawn to scale and the histogram for each ORF/region is presented in a different range of values.
(PDF)

## References

[1] Schiffman M, Wentzensen N (2013) Human papillomavirus infection and the multistage carcinogenesis of cervical cancer. Cancer Epidemiol Biomarkers Prev 22: 553-560. doi:10.1158/1055-9965.EPI-12-1406. PubMed: 23549399. 23549399 10.1158/1055-9965.EPI-12-1406 PMC3711590

[2] Jemal A, Bray F, Center MM, Ferlay J, Ward E et al. (2011) Global cancer statistics. CA Cancer J Clin 61: 69-90. doi:10.3322/caac.20107. PubMed: 21296855. 21296855 10.3322/caac.20107

[3] Forman D, de Martel C, Lacey CJ, Soerjomataram I, Lortet-Tieulent J et al. (2012) Global burden of human papillomavirus and related diseases. Vaccine 30 Suppl 5: F12-F23. doi:10.1016/j.vaccine.2012.07.055. PubMed: 23199955. 23199955 10.1016/j.vaccine.2012.07.055

[4] Bernard HU, Burk RD, Chen Z, van Doorslaer K, Hausen H et al. (2010) Classification of papillomaviruses (PVs) based on 189 PV types and proposal of taxonomic amendments. Virology 401: 70-79. doi:10.1016/j.virol.2010.02.002. PubMed: 20206957. 20206957 10.1016/j.virol.2010.02.002 PMC3400342

[5] de Villiers EM, Fauquet C, Broker TR, Bernard HU, zur Hausen H (2004) Classification of papillomaviruses. Virology 324: 17-27. doi:10.1016/j.virol.2004.03.033. PubMed: 15183049. 15183049 10.1016/j.virol.2004.03.033

[6] Muñoz N, Bosch FX, de Sanjosé S, Herrero R, Castellsagué X et al. (2003) Epidemiologic classification of human papillomavirus types associated with cervical cancer. N Engl J Med 348: 518-527. doi:10.1056/NEJMoa021641. PubMed: 12571259. 12571259 10.1056/NEJMoa021641

[7] Li N, Franceschi S, Howell-Jones R, Snijders PJ, Clifford GM (2011) Human papillomavirus type distribution in 30,848 invasive cervical cancers worldwide: Variation by geographical region, histological type and year of publication. Int J Cancer J Int Cancer 128: 927-935. doi:10.1002/ijc.25396. PubMed: 20473886. 10.1002/ijc.25396 20473886

[8] Smith JS, Lindsay L, Hoots B, Keys J, Franceschi S et al. (2007) Human papillomavirus type distribution in invasive cervical cancer and high-grade cervical lesions: a meta-analysis update. Int J Cancer 121: 621-632. doi:10.1002/ijc.22527. PubMed: 17405118. 17405118 10.1002/ijc.22527

[9] Bernard HU, Burk RD, de Villiers EM, zur Hausen H (2011) Papillomaviridae. In: King AM Adams MJ Lefkowitz E Virus taxonomy: ninth report of the International Committee on Taxonomy of Viruses. San Diego: Elsevier pp. 235-248.

[10] Burk RD, Chen Z, Harari A, Smith BC, Kocjan BJ et al. (2011) Classification and nomenclature system for Human Alphapapillomavirus variants: general features, nucleotide landmarks and assignment of HPV6 and HPV11 isolates to variant lineages. Acta Dermatovenerol Alp Panonica Adriat 20: 113-123 PubMed : 22131111 PMC3690374 22131111

[11] Smith B, Chen Z, Reimers L, van Doorslaer K, Schiffman M et al. (2011) Sequence imputation of HPV16 genomes for genetic association studies. PLOS ONE 6: e21375. doi:10.1371/journal.pone.0021375. PubMed: 21731721. 21731721 10.1371/journal.pone.0021375 PMC3121793

[12] Chen Z, Schiffman M, Herrero R, Desalle R, Anastos K et al. (2011). volution Taxonomic Classifications Hum Papillomavirus 16 (HPV16)-related variant genomes: HPV31, HPV33, HPV35, HPV52, HPV58 and HPV67. PLoS ONE 6: e20183

[13] Ho L, Chan SY, Burk RD, Das BC, Fujinaga K et al. (1993) The genetic drift of human papillomavirus type 16 is a means of reconstructing prehistoric viral spread and the movement of ancient human populations. J Virol 67: 6413-6423. PubMed: 8411343. 8411343 10.1128/jvi.67.11.6413-6423.1993 PMC238076

[14] Ong CK, Chan SY, Campo MS, Fujinaga K, Mavromara-Nazos P et al. (1993) Evolution of human papillomavirus type 18: an ancient phylogenetic root in Africa and intratype diversity reflect coevolution with human ethnic groups. J Virol 67: 6424-6431. PubMed: 8411344. 8411344 10.1128/jvi.67.11.6424-6431.1993 PMC238077

[15] Cornet I, Gheit T, Iannacone MR, Vignat J, Sylla BS et al. (2013) HPV16 genetic variation and the development of cervical cancer worldwide. Br J Cancer 108: 240-244. doi:10.1038/bjc.2012.508. PubMed: 23169278. 23169278 10.1038/bjc.2012.508 PMC3553516

[16] Chan PK, Zhang C, Park JS, Smith-McCune KK, Palefsky JM et al. (2013) Geographical distribution and oncogenic risk association of human papillomavirus type 58 E6 and E7 sequence variations. Int J Cancer 132: 2528-2536. doi:10.1002/ijc.27932. PubMed: 23136059. 23136059 10.1002/ijc.27932 PMC3962828

[17] Chen Z, Terai M, Fu L, Herrero R, DeSalle R et al. (2005) Diversifying selection in human papillomavirus type 16 lineages based on complete genome analyses. J Virol 79: 7014-7023. doi:10.1128/JVI.79.11.7014-7023.2005. PubMed: 15890941. 15890941 10.1128/JVI.79.11.7014-7023.2005 PMC1112126

[18] Chen Z, DeSalle R, Schiffman M, Herrero R, Burk RD (2009) Evolutionary dynamics of variant genomes of human papillomavirus types 18, 45, and 97. J Virol 83: 1443-1455. doi:10.1128/JVI.02068-08. PubMed: 19036820. 19036820 10.1128/JVI.02068-08 PMC2620887

[19] Arias-Pulido H, Peyton CL, Torrez-Martínez N, Anderson DN, Wheeler CM (2005) Human papillomavirus type 18 variant lineages in United States populations characterized by sequence analysis of LCR-E6, E2, and L1 regions. Virology 338: 22-34. doi:10.1016/j.virol.2005.04.022. PubMed: 15936050. 15936050 10.1016/j.virol.2005.04.022

[20] Godínez JM, Heideman DA, Gheit T, Alemany L, Snijders PJ et al. (2013) Differential presence of Papillomavirus variants in cervical cancer: an analysis for HPV33, HPV45 and HPV58. Infect Genet Evol 13: 96-104. doi:10.1016/j.meegid.2012.09.011. PubMed: 23022714. 23022714 10.1016/j.meegid.2012.09.011

[21] Alizon S, Luciani F, Regoes RR (2011) Epidemiological and clinical consequences of within-host evolution. Trends Microbiol 19: 24-32. doi:10.1016/j.tim.2010.09.005. PubMed: 21055948. 21055948 10.1016/j.tim.2010.09.005

[22] Xi LF, Kiviat NB, Hildesheim A, Galloway DA, Wheeler CM et al. (2006) Human papillomavirus type 16 and 18 variants: race-related distribution and persistence. J Natl Cancer Inst 98: 1045-1052. doi:10.1093/jnci/djj297. PubMed: 16882941. 16882941 10.1093/jnci/djj297

[23] Sichero L, Ferreira S, Trottier H, Duarte-Franco E, Ferenczy A et al. (2007) High grade cervical lesions are caused preferentially by non-European variants of HPVs 16 and 18. Int J Cancer 120: 1763-1768. doi:10.1002/ijc.22481. PubMed: 17230525. 17230525 10.1002/ijc.22481

[24] Hildesheim A, Schiffman M, Bromley C, Wacholder S, Herrero R et al. (2001) Human papillomavirus type 16 variants and risk of cervical cancer. J Natl Cancer Inst 93: 315-318. doi:10.1093/jnci/93.4.315. PubMed: 11181779. 11181779 10.1093/jnci/93.4.315

[25] Khouadri S, Villa LL, Gagnon S, Koushik A, Richardson H et al. (2006) Human papillomavirus type 33 polymorphisms and high-grade squamous intraepithelial lesions of the uterine cervix. J Infect Dis 194: 886-894. doi:10.1086/507431. PubMed: 16960775. 16960775 10.1086/507431

[26] Chan PK, Lam CW, Cheung TH, Li WW, Lo KW et al. (2002) Association of human papillomavirus type 58 variant with the risk of cervical cancer. J Natl Cancer Inst 94: 1249-1253. doi:10.1093/jnci/94.16.1249. PubMed: 12189229. 12189229 10.1093/jnci/94.16.1249

[27] Schiffman M, Rodriguez AC, Chen Z, Wacholder S, Herrero R et al. (2010) A population-based prospective study of carcinogenic human papillomavirus variant lineages, viral persistence, and cervical neoplasia. Cancer Res 70: 3159-3169. doi:10.1158/0008-5472.CAN-09-4179. PubMed: 20354192. 20354192 10.1158/0008-5472.CAN-09-4179 PMC2855741

[28] Xi LF, Schiffman M, Koutsky LA, Hulbert A, Lee SK et al. (2012) Association of human papillomavirus type 31 variants with risk of cervical intraepithelial neoplasia grades 2-3. Int J Cancer, 131: 2300–7. PubMed: 22396129. 22396129 10.1002/ijc.27520

[29] Herrero R, Castle PE, Schiffman M, Bratti MC, Hildesheim A et al. (2005) Epidemiologic profile of type-specific human papillomavirus infection and cervical neoplasia in Guanacaste, Costa Rica. J Infect Dis 191: 1796-1807. doi:10.1086/428850. PubMed: 15871111. 15871111 10.1086/428850

[30] Liaw K-L, Hsing AW, Chen C-J, Schiffman MH, Zhang TY et al. (1995) Human papillomavirus and cervical neoplasia: a case-control study in Taiwan. Int J Cancer 62: 565-571. doi:10.1002/ijc.2910620513. PubMed: 7665227. 7665227 10.1002/ijc.2910620513

[31] Wongworapat K, Keawvichit R, Sirirojn B, Dokuta S, Ruangyuttikarn C et al. (2008) Detection of human papillomavirus from self-collected vaginal samples of women in Chiang Mai, Thailand. Sex Transm Dis 35: 172-173. doi:10.1097/OLQ.0b013e318158af65. PubMed: 18216725. 18216725 10.1097/OLQ.0b013e318158af65

[32] Marks M, Gravitt PE, Gupta SB, Liaw KL, Kim E et al. (2011) The association of hormonal contraceptive use and HPV prevalence. Int J Cancer, 128: 2962–70. PubMed: 20734390. 20734390 10.1002/ijc.25628

[33] Singh DK, Anastos K, Hoover DR, Burk RD, Shi Q et al. (2009) Human Papillomavirus Infection and Cervical Cytology in HIV-Infected and HIV-Uninfected Rwandan Women. J Infect Dis 199: 1851-1861. doi:10.1086/599123. PubMed: 19435429. 19435429 10.1086/599123 PMC2814215

[34] Didelot-Rousseau MN, Nagot N, Costes-Martineau V, Vallès X, Ouedraogo A et al. (2006) Human papillomavirus genotype distribution and cervical squamous intraepithelial lesions among high-risk women with and without HIV-1 infection in Burkina Faso. Br J Cancer 95: 355-362. doi:10.1038/sj.bjc.6603252. PubMed: 16832413. 16832413 10.1038/sj.bjc.6603252 PMC2360631

[35] Sahasrabuddhe VV, Mwanahamuntu MH, Vermund SH, Huh WK, Lyon MD et al. (2007) Prevalence and distribution of HPV genotypes among HIV-infected women in Zambia. Br J Cancer 96: 1480-1483. PubMed: 17437020. 17437020 10.1038/sj.bjc.6603737 PMC2360194

[36] Wheeler CM, Yamada T, Hildesheim A, Jenison SA (1997) Human papillomavirus type 16 sequence variants: identification by E6 and L1 lineage-specific hybridization. J Clin Microbiol 35: 11-19. PubMed: 8968874. 8968874 10.1128/jcm.35.1.11-19.1997 PMC229505

[37] Terai M, Burk RD (2001) Characterization of a novel genital human papillomavirus by overlapping PCR: candHPV86 identified in cervicovaginal cells of a woman with cervical neoplasia. J Gen Virol 82: 2035-2040. PubMed: 11514712. 11514712 10.1099/0022-1317-82-9-2035

[38] Lurchachaiwong W, Junyangdikul P, Termrungruanglert W, Payungporn S, Sampatanukul P et al. (2010) Whole-genome sequence analysis of human papillomavirus type 18 from infected Thai women. Intervirology 53: 161-166. doi:10.1159/000274977. PubMed: 20068350. 20068350 10.1159/000274977

[39] Wu XL, Zhang CT, Zhu XK, Wang YC (2010) Detection of HPV types and neutralizing antibodies in women with genital warts in Tianjin City, China. Virol Sin 25: 8-17. doi:10.1007/s12250-010-3078-4. PubMed: 20960279. 20960279 10.1007/s12250-010-3078-4 PMC8227880

[40] Wu X, Zhang C, Feng S, Liu C, Li Y et al. (2009) Detection of HPV types and neutralizing antibodies in Gansu province, China. J Med Virol 81: 693-702. doi:10.1002/jmv.21435. PubMed: 19235880. 19235880 10.1002/jmv.21435

[41] Katoh K, Toh H (2010) Parallelization of the MAFFT multiple sequence alignment program. Bioinformatics 26: 1899-1900. doi:10.1093/bioinformatics/btq224. PubMed: 20427515. 20427515 10.1093/bioinformatics/btq224 PMC2905546

[42] Stamatakis A (2006) RAxML-VI-HPC: maximum likelihood-based phylogenetic analyses with thousands of taxa and mixed models. Bioinformatics 22: 2688-2690. doi:10.1093/bioinformatics/btl446. PubMed: 16928733. 16928733 10.1093/bioinformatics/btl446

[43] Guindon S, Gascuel O (2003) A simple, fast, and accurate algorithm to estimate large phylogenies by maximum likelihood. Syst Biol 52: 696-704. doi:10.1080/10635150390235520. PubMed: 14530136. 14530136 10.1080/10635150390235520

[44] Ronquist F, Huelsenbeck JP (2003) MrBayes 3: Bayesian phylogenetic inference under mixed models. Bioinformatics 19: 1572-1574. doi:10.1093/bioinformatics/btg180. PubMed: 12912839. 12912839 10.1093/bioinformatics/btg180

[45] Posada D, Crandall KA (1998) MODELTEST: testing the model of DNA substitution. Bioinformatics 14: 817-818. doi:10.1093/bioinformatics/14.9.817. PubMed: 9918953. 9918953 10.1093/bioinformatics/14.9.817

[46] Tamura K, Peterson D, Peterson N, Stecher G, Nei M et al. (2011) MEGA5: Molecular Evolutionary Genetics Analysis using Maximum Likelihood, Evolutionary Distance, and Maximum Parsimony Methods. Mol Biol Evol (submitted). PubMed: 21546353. 10.1093/molbev/msr121 PMC3203626 21546353

[47] Maddison DR, Maddison WP (2005) MacClade 4: Analysis of phylogeny and character evolution. 4.08 Sunderland MA Sinauer Associates. 10.1159/000156416 2606395

[48] Colwell RK, Chao A, Gotelli NJ, Lin S-Y, Mao CX et al. (2012) Models and estimators linking individual-based and sample-based rarefaction, extrapolation and comparison of assemblages. Plant Ecol 5: 3-21. doi:10.1093/jpe/rtr044.

[49] Delius H, Hofmann B (1994) Primer-directed sequencing of human papillomavirus types. Curr Top Microbiol Immunol 186: 13-31. doi:10.1007/978-3-642-78487-3_2. PubMed: 8205838. 8205838 10.1007/978-3-642-78487-3_2

[50] Rho J, Roy-Burman A, Kim H, de Villiers EM, Matsukura T et al. (1994) Nucleotide sequence and phylogenetic classification of human papillomavirus type 59. Virology 203: 158-161. doi:10.1006/viro.1994.1467. PubMed: 8030272. 8030272 10.1006/viro.1994.1467

[51] Reuter S, Delius H, Kahn T, Hofmann B, zur Hausen H et al. (1991) Characterization of a novel human papillomavirus DNA in the cervical carcinoma cell line ME180. J Virol 65: 5564-5568. PubMed: 1716694. 1716694 10.1128/jvi.65.10.5564-5568.1991 PMC249064

[52] Longuet M, Beaudenon S, Orth G (1996) Two novel genital human papillomavirus (HPV) types, HPV68 and HPV70, related to the potentially oncogenic HPV39. J Clin Microbiol 34: 738-744. PubMed: 8904450. 8904450 10.1128/jcm.34.3.738-744.1996 PMC228882

[53] Keinan A, Clark AG (2012) Recent explosive human population growth has resulted in an excess of rare genetic variants. Science 336: 740-743. doi:10.1126/science.1217283. PubMed: 22582263. 22582263 10.1126/science.1217283 PMC3586590

[54] Burk RD, Chen Z, Van Doorslaer K (2009) Human papillomaviruses: genetic basis of carcinogenicity. Public Health Genomics 12: 281-290. doi:10.1159/000214919. PubMed: 19684441. 19684441 10.1159/000214919 PMC2835381

[55] Chen Z, Fu L, Herrero R, Schiffman M, Burk RD (2007) Identification of a novel human papillomavirus (HPV97) related to HPV18 and HPV45. Int J Cancer 121: 2947-2952. 10.1002/ijc.22632 17351898

[56] Chen Z, van Doorslaer K, DeSalle R, Wood CE, Kaplan JR et al. (2009) Genomic diversity and interspecies host infection of alpha12 Macaca fascicularis papillomaviruses (MfPVs). Virology 393: 304-310. doi:10.1016/j.virol.2009.07.012. PubMed: 19716580. 19716580 10.1016/j.virol.2009.07.012 PMC3422072

[57] Rector A, Lemey P, Tachezy R, Mostmans S, Ghim SJ et al. (2007) Ancient papillomavirus-host co-speciation in Felidae. Genome Biol 8: R57. doi:10.1186/gb-2007-8-4-r57. PubMed: 17430578. 17430578 10.1186/gb-2007-8-4-r57 PMC1896010

