# Supplementary material for: Evolution and Taxonomic Classification of Alphapapillomavirus 7 Complete Genomes: HPV18, HPV39, HPV45, HPV59, HPV68 and HPV70
Source: PLoS One. 2013 Aug 16;8(8):e72565. doi: 10.1371/journal.pone.0072565 (PMC3745470; doi:10.1371/journal.pone.0072565)
Supplement: Table S1 — List, description and NCBI number of alpha-7 genomes. (PDF) [file pone.0072565.s001.pdf]

Table S1. Geographic origin of sample, lineage designation, length of complete genome and NCBI #.

Type	Isolate	Study	Lineage	Sublineage	NCBI Accession #	Length	GC%
HPV18	Ref		A	A1	AY262282	7857	40.44
HPV18	CU9		A	A1	GQ180785	7857	40.45
HPV18	Qv16302		A	A1	EF202144	7857	40.45
HPV18	CU12		A	A1	GQ180788	7857	40.41
HPV18	CU16		A	A1	GQ180792	7857	40.45
HPV18	CU15		A	A1	GQ180791	7856	40.43
HPV18	CU13		A	A1	GQ180789	7856	40.43
HPV18	CU8		A	A1	GQ180784	7857	40.42
HPV18	CU14		A	A1	GQ180790	7856	40.45
HPV18	Qv17052		A	A1	EF202145	7857	40.49
HPV18	Qv29226		A	A1	**KC470208**	7857	40.41
HPV18	Qv12247		A	A1	**KC470209**	7857	40.41
HPV18	Qv03132		A	A1	EF202143	7857	40.44
HPV18	Qv16306		A	A2	EF202146	7857	40.38
HPV18	Qv32981		A	A2	**KC470210**	7857	40.38
HPV18	Qv28978		A	A2	**KC470211**	7857	40.38
HPV18	Qv15586		A	A3	EF202147	7857	40.41
HPV18	Qv21751		A	A3	EF202148	7857	40.38
HPV18	Qv26861		A	A3	**KC470212**	7857	40.32
HPV18	Qv15957		A	A3	EF202149	7857	40.32
HPV18	CU10		A	A3	GQ180786	7856	40.39
HPV18	Qv02876		A	A4	EF202151	7857	40.33
HPV18	Qv17955		A	A4	EF202150	7857	40.40
HPV18	Z135		A	A4	**KC470213**	7857	40.35
HPV18	CU11		A	A5	GQ180787	7844	40.29
HPV18	Z52		B	B1	**KC470214**	7824	40.01
HPV18	Z63		B	B1	**KC470215**	7824	40.07
HPV18	Z53		B	B1	**KC470216**	7824	40.03
HPV18	Rw750		B	B1	**KC470217**	7824	40.04
HPV18	Rw687		B	B1	**KC470218**	7824	40.07
HPV18	Rw57		B	B1	**KC470219**	7824	40.08
HPV18	Rw830		B	B1	**KC470220**	7824	40.08
HPV18	Qv03814		B	B1	EF202154	7824	40.11
HPV18	Qv04924		B	B1	EF202155	7824	40.12
HPV18	Qv21444		B	B1	EF202153	7824	40.16
HPV18	Qv28775		B	B1	**KC470221**	7824	40.09
HPV18	Z100		B	B1	**KC470222**	7824	40.08
HPV18	BF309		B	B2	**KC470223**	7824	40.07
HPV18	BF288		B	B2	**KC470224**	7824	40.07
HPV18	BF172		B	B2	**KC470225**	7824	40.07
HPV18	Qv17199		B	B3	EF202152	7844	40.06
HPV18	Z125		B	B3	**KC470226**	7844	40.01
HPV18	Qv12693		B	B3	**KC470227**	7844	39.98
HPV18	BF380		B	B3	**KC470228**	7844	40.01
HPV18	Qv39775		C		**KC470229**	7837	40.14
HPV18	BF226		C		**KC470230**	7837	40.05
HPV39	Ref		A	A1	M62849	7833	40.15
HPV39	Qv28005		A	A1	**KC470231**	7833	40.16
HPV39	Qv18075		A	A1	**KC470232**	7833	40.16
HPV39	Qv29613		A	A1	**KC470233**	7833	40.11
HPV39	Qv29778		A	A1	**KC470234**	7833	40.14
HPV39	Qv31864		A	A1	**KC470235**	7833	40.14
HPV39	Qv29509		A	A1	**KC470236**	7885	40.09
HPV39	Qv27715		A	A1	**KC470237**	7833	40.13
HPV39	Qv36565		A	A1	**KC470238**	7833	40.07
HPV39	Qv21219		A	A2	**KC470239**	7860	40.27
HPV39	Qv25959		A	A2	**KC470240**	7860	40.34
HPV39	Qv25984		A	A2	**KC470241**	7860	40.23
HPV39	Qv25609		A	A2	**KC470242**	7860	40.28
HPV39	Tw562		A	A2	**KC470243**	7860	40.33
HPV39	As093		A	A2	**KC470244**	7860	40.36
HPV39	Rw20		A	A2	**KC470245**	7860	40.23
HPV39	Rw15		A	A2	**KC470246**	7860	40.23
HPV39	Rw72		B		**KC470247**	7833	40.24
HPV39	BF182		B		**KC470248**	7833	40.24
HPV39	BF375		B		**KC470249**	7833	40.25

Table S1. continue

Type	Isolate	Study	Lineage	Sublineage	NCBI Accession #	Length	GC%
HPV45	Ref		A	A1	X74479	7858	39.63
HPV45	Qv20214		A	A1	EF202156	7858	39.63
HPV45	Z79		A	A1	**KC470250**	7858	39.60
HPV45	Z5		A	A1	**KC470251**	7858	39.62
HPV45	RW632		A	A1	**KC470252**	7849	39.57
HPV45	Qv02356		A	A2	**KC470253**	7848	39.65
HPV45	Qv27648		A	A2	**KC470254**	7848	39.70
HPV45	BF208		A	A2	**KC470255**	7848	39.73
HPV45	Qv27565		A	A2	EF202157	7848	39.72
HPV45	Qv30004		A	A2	EF202160	7848	39.69
HPV45	Qv33330		A	A2	EF202158	7848	39.68
HPV45	Qv34178		A	A2	EF202159	7848	39.69
HPV45	BF134		A	A3	**KC470256**	7841	39.64
HPV45	Qv00550		B	B1	EF202161	7849	39.74
HPV45	Qv06560		B	B1	EF202163	7841	39.79
HPV45	Qv35960		B	B1	EF202162	7849	39.76
HPV45	RW894		B	B1	**KC470257**	7849	39.76
HPV45	RW46		B	B2	**KC470258**	7849	39.70
HPV45	Qv30712		B	B2	**KC470259**	7848	39.76
HPV45	Qv25000		B	B2	EF202164	7849	39.75
HPV45	Qv34163		B	B2	**KC470260**	7848	39.73
HPV45	Qv31748		B	B2	EF202167	7849	39.74
HPV45	Qv26351		B	B2	EF202165	7849	39.75
HPV45	Qv31035		B	B2	EF202166	7849	39.71
HPV59	Ref		A	A1	X77858	7896	38.64
HPV59	LZod68		A	A1	EU918767	7897	38.82
HPV59	Qv25652		A	A2	**KC470261**	7898	38.67
HPV59	Qv00231		A	A2	**KC470262**	7898	38.68
HPV59	Qv23880		A	A3	**KC470263**	7897	38.69
HPV59	Qv25808		B		**KC470264**	7898	38.79
HPV59	Qv33993		B		**KC470265**	7898	38.78
HPV59	Qv33361		B		**KC470266**	7898	38.78
HPV68	Ref		A	A1	DQ080079	7822	40.12
HPV68	Qv23347		A	A1	**KC470267**	7822	40.10
HPV68	Qv32718		A	A1	**KC470268**	7822	40.10
HPV68	Qv01017		A	A2	**KC470269**	7822	40.09
HPV68	Qv18016		B		**KC470270**	7814	40.09
HPV68	Qv30759		B		**KC470271**	7814	40.09
HPV68(ME180)	Ref		C	C1	FR751039	7836	39.96
HPV68	Qv24962		C	C1	**KC470272**	7836	39.87
HPV68	Qv19111		C	C1	**KC470273**	7836	39.83
HPV68	Qv33015		C	C2	**KC470274**	7836	39.71
HPV68	LZod68		C	C2	EU918769	7834	39.83
HPV68	Qv00677		D	D1	**KC470275**	7830	39.74
HPV68	Qv30285		D	D2	**KC470276**	7830	39.87
HPV68	Qv17725		E		**KC470277**	7830	39.78
HPV68	TJ42		E		GQ472851	7830	40.00
HPV68	Qv30698		E		**KC470278**	7830	39.90
HPV68	Qv25395		F	F1	**KC470279**	7828	40.07
HPV68	Qv17231		F	F2	**KC470280**	7836	39.93
HPV68	Rw826		F	F2	**KC470281**	7838	39.83
HPV68	Rw900		F	F2	**KC470282**	7836	39.87
HPV68	Qv33999		F	F2	**KC470283**	7836	39.82
HPV70	Ref		A		U21941	7905	40.33
HPV70	Qv27542		A		**KC470284**	7905	40.33
HPV70	Qv27211		A		**KC470285**	7905	40.29
HPV70	Qv28219		A		**KC470286**	7910	40.33
HPV70	Qv05102		B		**KC470287**	7922	40.25
HPV70	Qv33565		B		**KC470288**	7918	40.30
HPV70	QV35039		B		**KC470289**	7922	40.25
HPV70	Qv27399		B		**KC470290**	7922	40.27
HPV70	Qv17574		B		**KC470291**	7922	40.22
HPV85	Ref		A		AF131950	7812	37.71
HPV97	Ref		A		EF202168	7843	37.42
