# Supplementary material for: Evolution and Taxonomic Classification of Alphapapillomavirus 7 Complete Genomes: HPV18, HPV39, HPV45, HPV59, HPV68 and HPV70
Source: PLoS One. 2013 Aug 16;8(8):e72565. doi: 10.1371/journal.pone.0072565 (PMC3745470; doi:10.1371/journal.pone.0072565)
Supplement: Table S2 — Variability of alpha-7 HPV genome regions and open reading frames (ORFs). (PDF) [file pone.0072565.s002.pdf]

Table S2. Variation of alpha-7 HPV genome regions and open reading frames (ORFs).

Type/Gene	Max nt pairwise difference	Number of nt sequences	Number of variable nt positions [a]		Variable nt codon positions [b]			Max aa pairwise difference	Number of aa sequences [c]	Number of variable aa positions [d]	
			no.	%	1st	2nd	3rd			no.	%
**HPV18 (N=46)**											
E6	2.3%	477	16	3.4%	3	2	11	1.9%	158	5	3.2%
E7	2.2%	318	14	4.4%	4	2	8	3.8%	105	5	4.8%
E1	1.9%	1974	83	4.2%	20	10	53	1.5%	657	28	4.3%
E2	2.6%	1098	61	5.6%	16	16	28	4.4%	365	34	9.3%
E4	3.1%	267	18	6.7%	4	7	6	9.4%	88	14	15.9%
NCR1											
E5	3.6%	222	14	6.3%	7	3	4	8.2%	73	10	13.7%
NCR2	8.1%	86	10	11.6%							
L2	3.2%	1389	101	7.3%	20	15	66	4.1%	462	37	8.0%
L1	1.6%	1524	65	4.3%	13	11	41	1.8%	507	23	4.5%
URR	3.3%	825	59	7.2%							
CG [e]	2.1%	7857	422	5.4%				2.4%	2415	156	6.5%
**HPV39 (N=20)**											
E6	1.5%	477	9	1.9%	2	4	3	3.8%	158	7	4.4%
E7	1.8%	330	8	2.4%	5	0	3	2.8%	109	4	3.7%
E1	0.8%	1971	38	1.9%	10	4	23	0.9%	650	11	1.7%
E2	1.0%	1113	20	1.8%	7	2	11	1.6%	370	10	2.7%
E4	1.4%	285	5	1.8%	0	2	3	2.1%	94	2	2.1%
NCR1											
E5	2.7%	219	7	3.2%	3	1	3	5.6%	72	5	6.9%
NCR2	4.3%	47	3	6.4%							
L2	1.5%	1413	39	2.8%	5	7	27	1.3%	470	13	2.8%
L1	1.0%	1518	27	1.8%	8	3	16	1.0%	505	9	1.8%
URR	2.4%	831	34	4.1%							
CG [e]	1.1%	7912	186	2.4%				1.1%	2428	61	2.5%
**HPV45 (N=24)**											
E6	1.9%	477	14	2.9%	4	5	5	4.4%	158	8	5.1%
E7	2.5%	321	10	3.1%	2	2	6	5.7%	106	7	6.6%
E1	1.0%	1932	37	1.9%	9	3	25	1.2%	643	14	2.2%
E2	2.2%	1107	37	3.3%	13	8	16	3.8%	368	20	5.4%
E4	2.9%	273	11	4.0%	3	2	6	6.7%	90	8	8.9%
NCR1	6.10%	33	2	6.1%							
E5	1.8%	222	4	1.8%	2	0	2	2.7%	73	2	2.7%
NCR2	6.7%	105	11	10.5%							
L2	1.9%	1392	48	3.4%	11	8	29	2.8%	463	20	4.3%
L1	1.3%	1542	33	2.1%	3	5	24	1.2%	513	10	1.9%
URR	2.6%	810	45	5.6%							
CG [e]	1.5%	7858	239	3.0%				1.8%	2414	89	3.7%
**HPV59 (N=8)**											
E6	1.5%	483	11	2.3%	4	2	5	1.9%	160	4	2.5%
E7	0.9%	234	3	1.3%	1	1	1	2.8%	107	3	2.8%
E1	1.0%	1935	28	1.4%	9	2	17	1.1%	644	8	1.2%
E2	1.3%	1113	21	1.9%	7	8	6	3.2%	370	17	4.6%
E4	2.0%	348	11	3.2%	4	1	6	1.7%	115	2	1.7%
NCR1											
E5	2.3%	222	6	2.7%	5	1	0	6.8%	73	6	8.2%
NCR2	3.0%	101	5	5.0%							
L2	2.1%	1395	43	3.1%	7	5	31	2.2%	464	13	2.8%
L1	1.3%	1527	29	1.9%	6	5	18	1.8%	508	13	2.6%
URR	1.7%	822	20	2.4%							
CG [e]	1.3%	7898	169	2.1%				1.7%	2441	66	2.7%

Table S2. continue

Type/Gene	Max nt pairwise difference	Number of nt sequences	Number of variable nt positions [a]		Variable nt codon positions [b]			Max aa pairwise difference	Number of aa sequences [c]	Number of variable aa positions [d]	
			no.	%	1st	2nd	3rd			no.	%
**HPV68 (N=21)**											
E6	6.7%	477	44	9.2%	10	9	25	8.9%	158	17	10.8%
E7	5.4%	333	21	6.3%	5	8	8	10.9%	110	12	10.9%
E1	4.8%	1923	144	7.5%	43	11	90	4.2%	640	51	8.0%
E2	6.4%	1113	103	9.3%	36	17	50	9.7%	370	53	14.3%
E4	8.4%	285	29	10.2%	9	6	14	13.8%	94	16	17.0%
NCR1	13.3%	76	10	13.2%							
E5	13.5%	222	39	17.6%	16	6	17	23.3%	73	18	24.7%
NCR2	19.6%	46	10	21.7%							
L2	7.2%	1410	142	10.1%	32	7	103	5.3%	469	37	7.9%
L1	7.8%	1518	156	10.3%	29	11	116	6.1%	505	37	7.3%
URR	9.4%	818	105	12.8%							
CG [e]	6.7%	7850	771	9.8%				6.6%	2419	232	9.6%
**HPV70 (N=9)**											
E6	2.1%	477	15	3.1%	7	5	3	4.4%	158	11	7.0%
E7	1.5%	330	5	1.5%	1	2	2	3.7%	109	4	3.7%
E1	2.0%	1959	51	2.6%	13	8	30	1.5%	652	16	2.5%
E2	1.6%	1083	21	1.9%	4	6	11	2.2%	360	11	3.1%
E4	2.0%	255	7	2.7%	3	2	2	6.0%	84	5	6.0%
NCR1											
E5	2.6%	237	8	3.4%	3	3	2	5.4%	78	6	7.7%
NCR2	4.8%	70	4	5.7%							
L2	1.6%	1401	31	2.2%	4	4	23	0.9%	466	7	1.5%
L1	1.2%	1521	24	1.6%	3	1	19	1.0%	506	7	1.4%
URR	2.4%	911	33	3.6%							
CG [e]	1.6%	7922	192	2.4%				1.6%	2413	67	2.8%

[a] The number of nucleotides within each ORF and region of the genome based on one genome size for each HPV type calculated from the lobal sequence alignments. Range of values and location in the papillomavirus genome are shown in supplemental figure S4;
[b] Total number and percentage of positions based on one genome size for each HPV type calculated from the global sequence alignments. Nucleotide variations include SNPs and indels;
[c] Maximum number of encoded amino acids for each ORF;
[d] Total number and percentage of variable amino acids based on the maximum number of amino acids derived from the established genome size for each HPV type;
[e] CG, complete genome. Each nucleotide position is counted once. A single genome size is inferred from the nucleotide alignment (indel was treated as one event).
NCR1, non-coding region 1 (between E2 and E5 ORFs); NCR2, non-coding region 2 (between E5 and L2 ORFs); URR, up stream regulatory region (between stop codon of L1 and start codon of E6); CG, complete genome
