# Supplementary material for: Evolution and Taxonomic Classification of Alphapapillomavirus 7 Complete Genomes: HPV18, HPV39, HPV45, HPV59, HPV68 and HPV70
Source: PLoS One. 2013 Aug 16;8(8):e72565. doi: 10.1371/journal.pone.0072565 (PMC3745470; doi:10.1371/journal.pone.0072565)
Supplement: Table S3 — Comparison of nucleotide sequence differences between variant lineages and sublineages for each type. The intra-lineage (e.g., A vs. A) and intra-sublineage (e.g., A1 vs. A1) difference values are highlighted in gray. (PDF) [file pone.0072565.s003.pdf]

Table S3. Nucleotide sequence mean difference (± standard error) of alpha-7 HPV complete genomes. The intra-lineage (e.g., A vs. A) and intra-sublineage (e.g., A1 vs. A1) difference values are highlighted in gray.

**HPV18**	A1	A2	A3	A4	A5	B1	B2	B3	C
A1	0.13±0.02								
A2	0.37±0.06	0.05±0.02							
A3	0.47±0.06	0.45±0.07	0.25±0.04						
A4	0.52±0.07	0.50±0.07	0.40±0.06	0.14±0.04					
A5	0.83±0.10	0.82±0.09	0.75±0.08	0.73±0.09	n/c				
B1	1.93±0.15	1.89±0.15	1.87±0.16	1.91±0.16	1.78±0.16	0.20±0.03			
B2	1.97±0.15	1.93±0.15	1.91±0.16	1.95±0.16	1.80±0.16	0.35±0.05	0.13±0.03		
B3	1.95±0.16	1.91±0.15	1.88±0.17	1.92±0.16	1.81±0.16	0.42±0.07	0.41±0.06	0.10±0.03	
C	1.76±0.14	1.76±0.13	1.73±0.14	1.73±0.14	1.69±0.14	1.07±0.10	1.05±0.11	1.04±0.10	0.19±0.05

**HPV39**	A1	A2	B
A1	0.19±0.03		
A2	0.48±0.06	0.24±0.04	
B	0.98±0.09	0.97±0.08	0.35±0.05

**HPV45**	A1	A2	A3	B1	B2
A1	0.20±0.03				
A2	0.66±0.07	0.09±0.02			
A3	0.57±0.08	0.80±0.08	n/c		
B1	1.21±0.12	1.37±0.11	1.19±0.12	0.16±0.04	
B2	1.23±0.11	1.33±0.11	1.21±0.12	0.72±0.08	0.10±0.02

**HPV59**	A1	A2	A3	B
A1	0.41±0.07			
A2	0.60±0.08	0.13±0.04		
A3	0.89±0.10	0.82±0.11	n/c	
B	1.26±0.12	1.11±0.11	1.14±0.11	0.04±0.02

**HPV68**	A1	A2	B	C1	C2	D1	D2	E	F1	F2
A1	0.22±0.04									
A2	0.46±0.06	n/c								
B	0.93±0.09	0.97±0.09	0.23±0.05							
C1	6.45±0.28	6.50±0.29	6.53±0.26	0.26±0.04						
C2	6.43±0.27	6.48±0.27	6.53±0.26	0.49±0.07	0.33±0.07					
D1	6.49±0.27	6.51±0.27	6.51±0.27	1.56±0.12	1.47±0.12	n/c				
D2	6.47±0.26	6.50±0.27	6.50±0.27	1.54±0.14	1.46±0.14	0.49±0.08	n/c			
E	6.54±0.27	6.60±0.27	6.61±0.27	1.72±0.11	1.60±0.12	0.91±0.08	0.94±0.10	0.35±0.05		
F1	6.38±0.27	6.43±0.27	6.45±0.27	1.40±0.13	1.28±0.11	1.34±0.13	1.29±0.13	1.50±0.11	n/c	
F2	6.39±0.28	6.44±0.28	6.46±0.28	1.31±0.12	1.20±0.11	1.25±0.12	1.25±0.14	1.45±0.12	0.77±0.08	0.35±0.04

**HPV70**	A	B
A	0.32±0.04	
B	1.52±0.13	0.36±0.05
