# Supplementary material for: Evolution and Taxonomic Classification of Alphapapillomavirus 7 Complete Genomes: HPV18, HPV39, HPV45, HPV59, HPV68 and HPV70
Source: PLoS One. 2013 Aug 16;8(8):e72565. doi: 10.1371/journal.pone.0072565 (PMC3745470; doi:10.1371/journal.pone.0072565)
Supplement: Figure S1 — Sequence polymorphisms at nucleotide and amino acid positions within the complete genomes and ORFs of alpha-7 HPV variants. Amino acids alignments were used to guide the nucleotide sequence alignments. The original GenBank sequence for each type is used as the reference for all alignments and is shown at the top of each panel. Only sites that are different from the reference sequence in one or more of the isolates (name is on the left of the panel with the type|sample identifier|lineage or sublineage listed) are displayed. Below the nucleotide sequence alignments are the corresponding amino acid differences for each ORF. The nucleotide sequence variations are shown for each position listed at the top of the panel by ORF or region. Under the reference sequence the nucleotide sequence of each isolate is displayed listing only sites that are different. Dots, sites matched with reference sequence; dashes, indel events. NCR1, noncoding region between E2 and E5 ORFs; NCR2, noncoding region between E5 and L2 ORFs; URR, upstream regulatory region located between stop codon of L1 and start codon of E6. Genome sequences for each lineage or sublineage are alternatively shown as grey blocks for visualization of most closely related isolates: (A) alignment of HPV18 complete genomes, (B) alignment of HPV39 complete genomes, (C) alignment of HPV45 complete genomes, (D) alignment of HPV59 complete genomes, (E) alignment of HPV68 complete genomes, and (F) alignment of HPV70 complete genomes. (PDF) [file pone.0072565.s004.pdf]

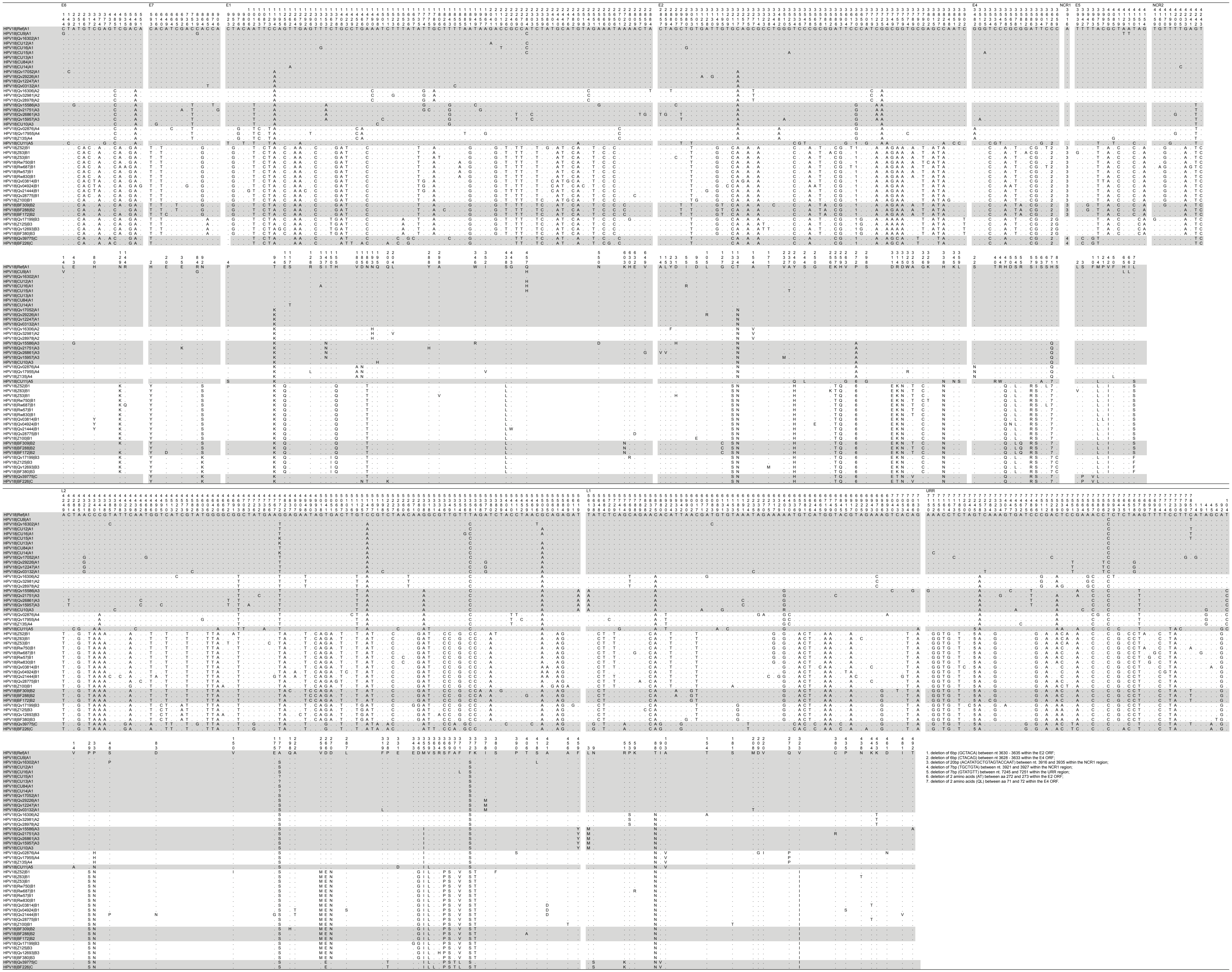

1. deletion of 6bp (GCTACA) between nt 3630 - 3635 within the E2 ORF;
2. deletion of 6bp (CTACAG) between nt 3628 - 3633 within the E4 ORF;
3. deletion of 20bp (ACATATGCTGTAGTACCAAT) between nt. 3916 and 3935 within the NCR1 region;
4. deletion of 7bp (TGCTGTA) between nt. 3921 and 3927 within the NCR1 region;
5. deletion of 7bp (GTATGTT) between nt. 7245 and 7251 within the URR region;
6. deletion of 2 amino acids (AT) between aa 272 and 273 within the E2 ORF;
7. deletion of 2 amino acids (QL) between aa 71 and 72 within the E4 ORF.

Fig. S1A

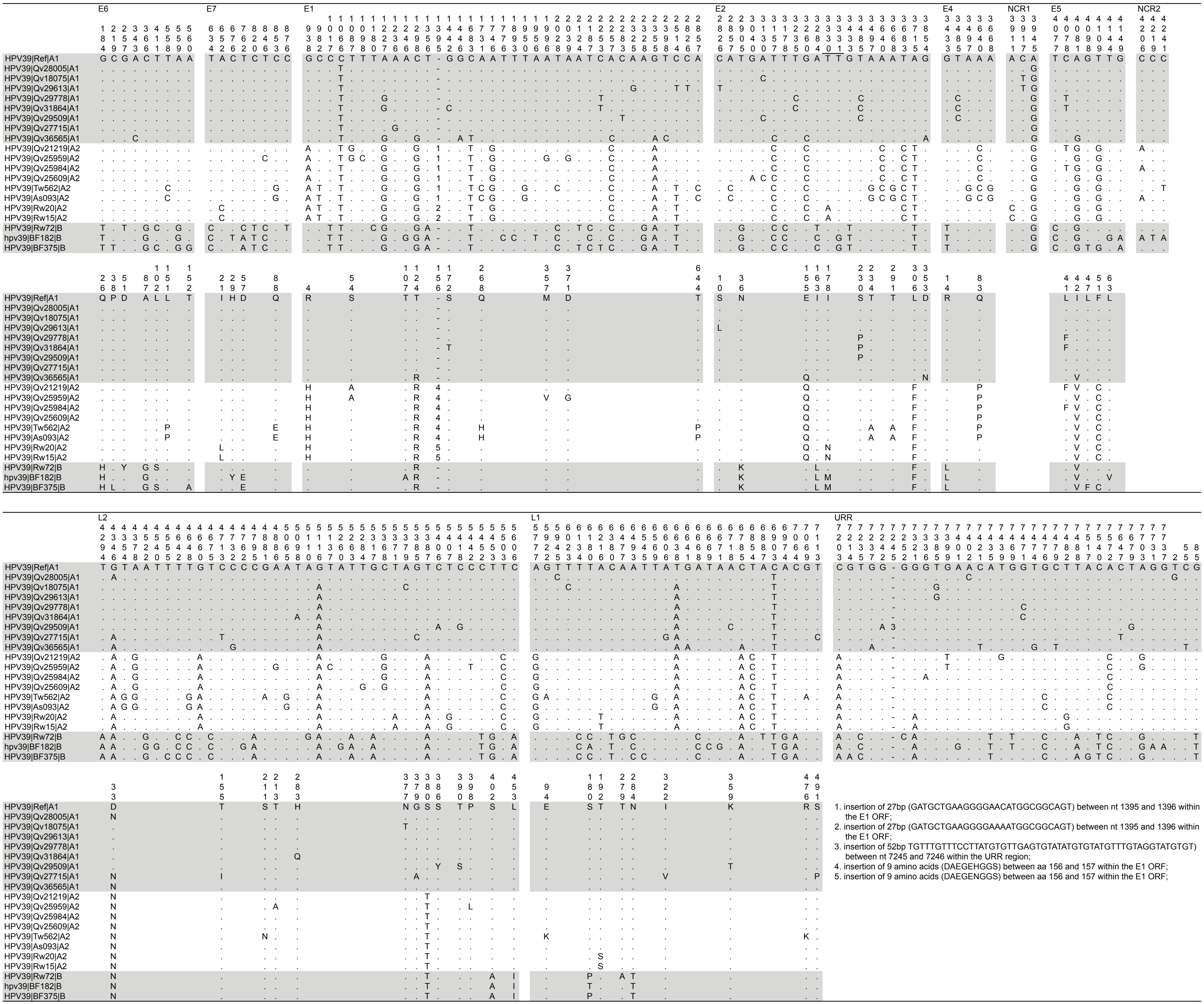

1. insertion of 27bp (GATGCTGAAGGGGAACATGGCGGCAGT) between nt 1395 and 1396 within the E1 ORF;
2. insertion of 27bp (GATGCTGAAGGGGAAAATGGCGGCAGT) between nt 1395 and 1396 within the E1 ORF;
3. insertion of 52bp TGTTTGTTTCCTTATGTGTTGAGTGTATATGTGTATGTTTGTAGGTATGTGT) between nt 7245 and 7246 within the URR region;
4. insertion of 9 amino acids (DAEGEHGGS) between aa 156 and 157 within the E1 ORF;
5. insertion of 9 amino acids (DAEGENGGS) between aa 156 and 157 within the E1 ORF;


Fig. S1B

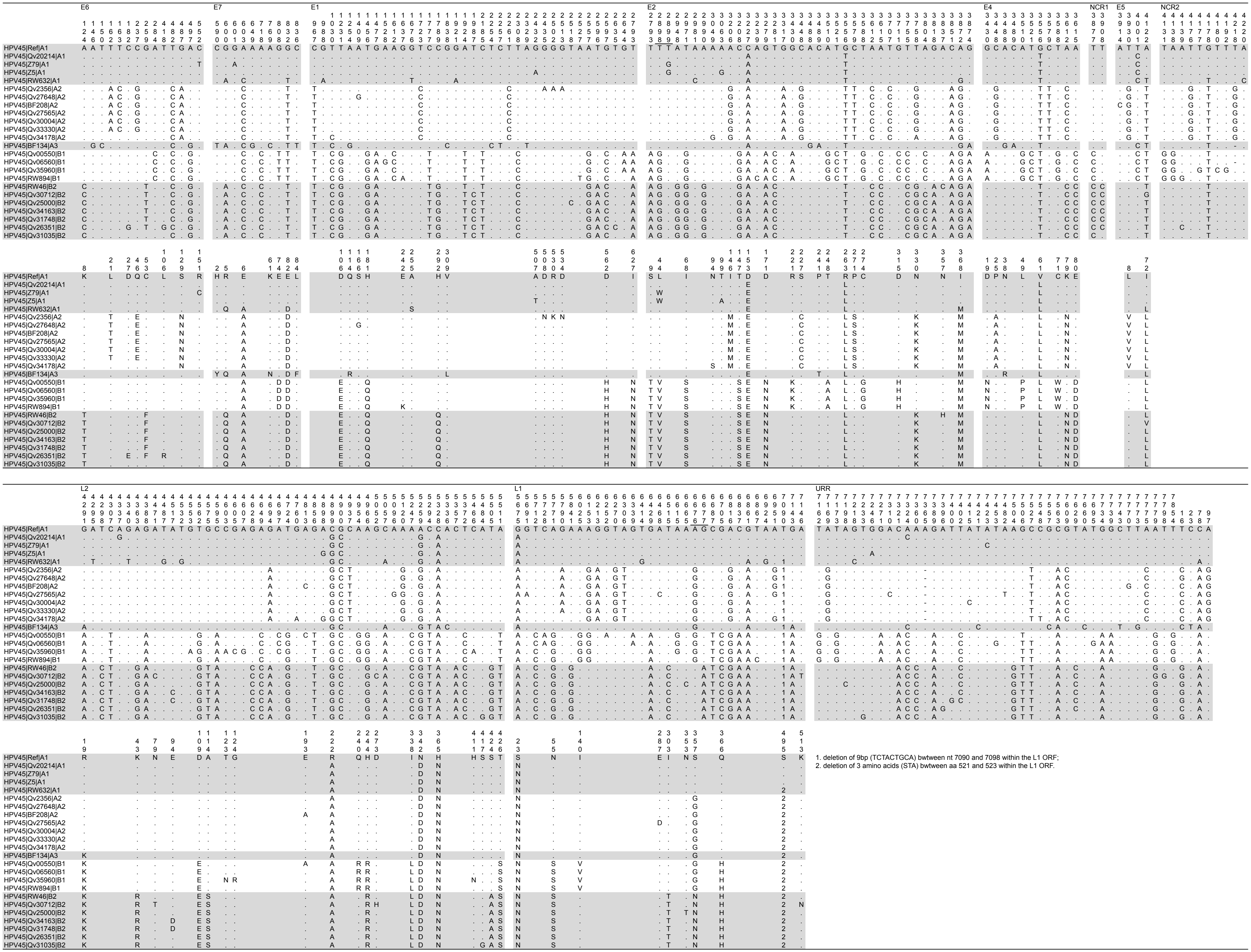


1. deletion of 9bp (TCTACTGCA) bwtween nt 7090 and 7098 within the L1 ORF;
2. deletion of 3 amino acids (STA) bwtween aa 521 and 523 within the L1 ORF.

Fig. S1C

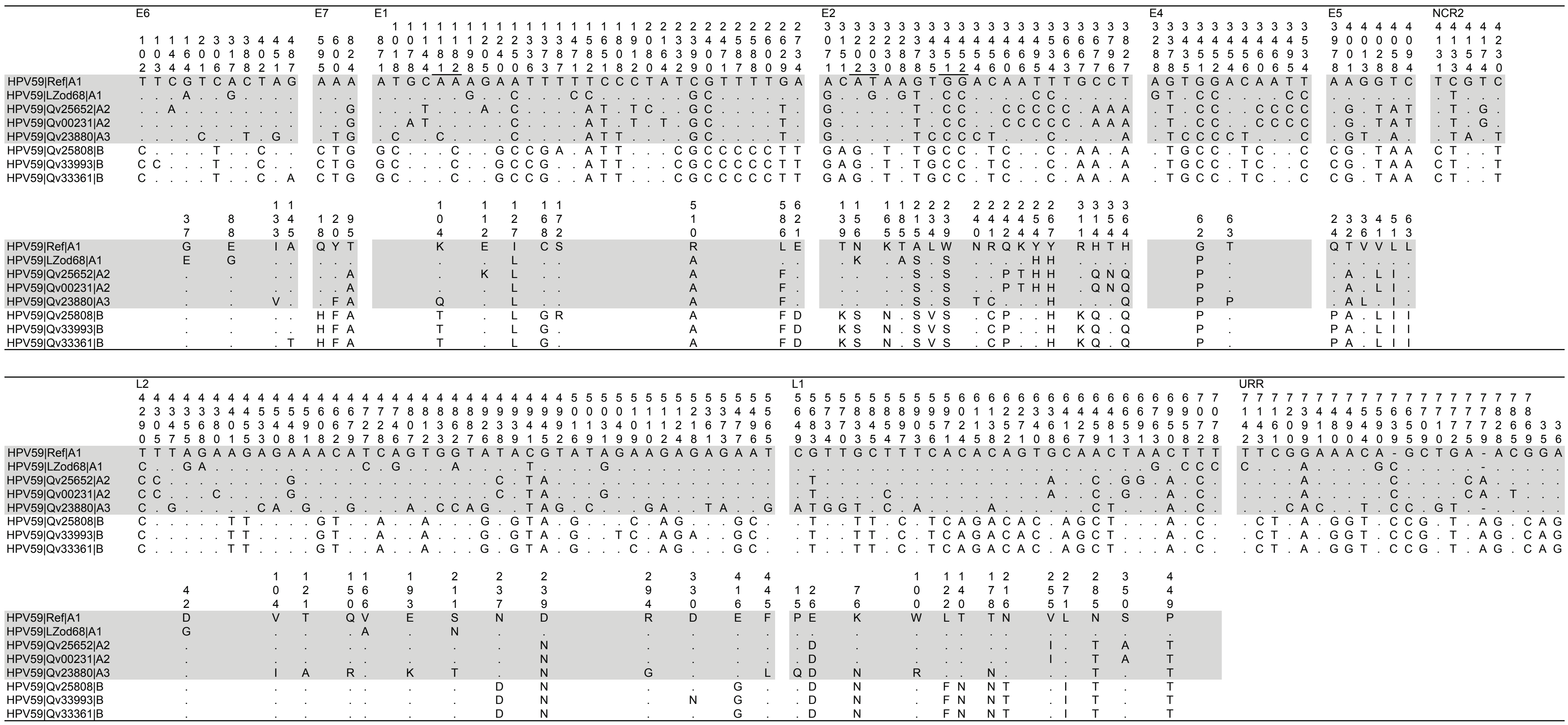
E6
E7
E1
E2
E4
E5
NCR2
L2
L1
URR
HPV59|Ref|A1
HPV59|LZod68|A1
HPV59|Qv25652|A2
HPV59|Qv00231|A2
HPV59|Qv23880|A3
HPV59|Qv25808|B
HPV59|Qv33993|B
HPV59|Qv33361|B

Fig. S1D

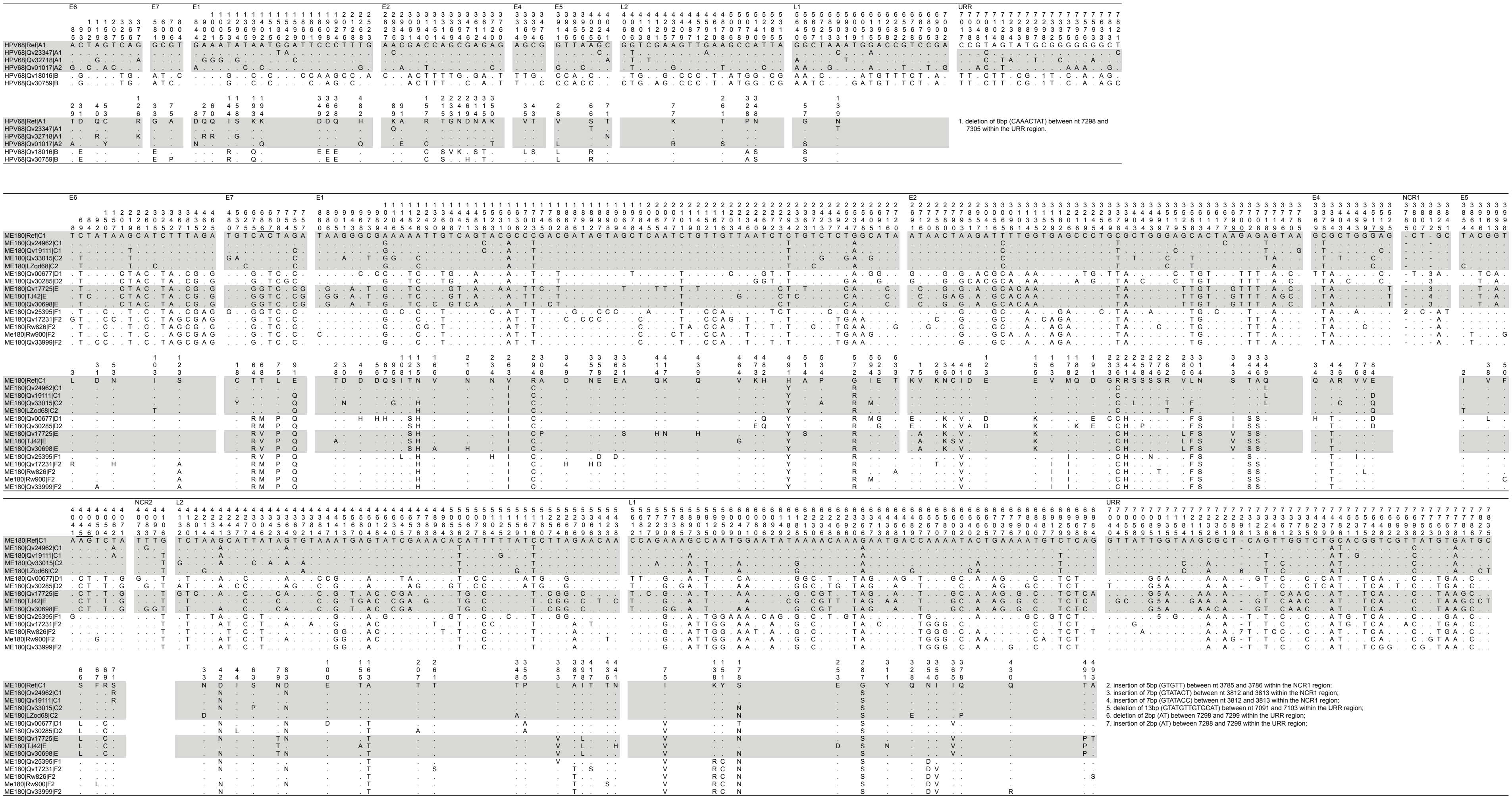
E6
E7
E1
E2
E4
E5
L2
L1
URR
NCR1
NCR2
1. deletion of 8bp (CAAACTAT) between nt 7298 and 7305 within the URR region.
2. insertion of 5bp (GTGTT) between nt 3785 and 3786 within the NCR1 region;
3. insertion of 7bp (GTATACT) between nt 3812 and 3813 within the NCR1 region;
4. insertion of 7bp (GTATACC) between nt 3812 and 3813 within the NCR1 region;
5. deletion of 13bp (GTATGTTGTGCAT) between nt 7091 and 7103 within the URR region;
6. deletion of 2bp (AT) between 7298 and 7299 within the URR region;
7. insertion of 2bp (AT) between 7298 and 7299 within the URR region;

Fig. S1E

| | E6 | E7 | E1 | E2 | E4 |
|---|---|---|---|---|---|
| Position (nt) | 120, 125, 241, 175, 213, 264, 286, 320, 335, 344, 353, 404, 487, 491, 492 | 613, 621, 707, 840, 896 | 939, 1023, 1056, 1070, 1092, 1122, 1155, 1220, 1366, 1470, 1524, 1540, 1542, 1599, 1626, 1677, 1727, 1759, 1765, 1772, 1794, 1866, 1937, 2007, 2184, 2218, 2269, 2271, 2274, 2287, 2331, 2340, 2391, 2413, 2457, 2476, 2529, 2543, 2554, 2556, 2631, 2637, 2647, 2662, 2678, 2682, 2691, 2739, 2766, 2789, 2794 | 2917, 3163, 3171, 3262, 3275, 3319, 3340, 3349, 3373, 3503, 3561, 3562, 3568, 3587, 3591, 3618, 3721, 3722, 3751, 3810, 3849 | 3503, 3561, 3562, 3568, 3587, 3591, 3618 |
| HPV70\|Ref\|A | CCATATGCGCAATCA | TGCAC | TGACAAACGTACAGCAACTGGACTAACAGGCGTTTGCGGTCCGCGATCTAG | CATGGGGGCGTCAGTGTATCG | GTCAGTG |
| HPV70\|Qv27542\|A | T..........G... | ..... | ................................C...T.............. | ...................A. | ....... |
| HPV70\|Qv27211\|A | .T.........G... | ..... | .............T.....A............C...T.............. | ..................GA. | ....... |
| HPV70\|Qv28219\|A | ...........G... | ..... | ..TG.GG....A.......AA.A.........C...T.............. | .........A..G......A. | A..G... |
| HPV70\|Qv5102\|B | A.TG.GA...GGCAG | GTGG. | ........TCGAG.TC.TCAA..CG.TGA.T..C.....CT....G.TCG. | AGAACA.TT..TG.CTCCCAA | ..TG.CT |
| HPV70\|Qv33565\|B | A.....AT..GG.AG | GTGG. | .......T.CGAG.TC.TC.A..CG.TGA.T..C.....CT....G.TCG. | .G.ACA.TT..TGCCTCCCAA | ..TGCCT |
| HPV70\|QV35039\|B | T.....AT..GG.AG | GTGG. | ....T....CGAG.TC.TCAA..CG.TGA.T..C.....CT.AT.G.TCG. | .G.ACA.TT..TGCCTCCCAA | ..TGCCT |
| HPV70\|Qv27399\|B | .T....A...GGCAG | GTGGT | ........TCGAG.TCTTCAAG.CGTTGACTA.CC....CG.A.AGCTC.. | .G.ACATTT.CTG.CTCCCAA | .CTG.CT |
| HPV70\|Qv17574\|B | ....C.ATCTGGCAG | GTGG. | CA......TCGAG.TC.TC.A..CG.TGA.TA.CCA.AACTTA.AG.TC.A | .G.A.ATTT.CTG.CTCCCAA | .CTG.CT |

| | E6 | E7 | E1 | E2 | E4 |
|---|---|---|---|---|---|
| Position (aa) | 5, 7, 12, 36, 53, 72, 77, 80, 83, 100, 129 | 8, 10, 39, 102 | 48, 98, 147, 205, 267, 282, 337, 454, 517, 539, 543, 574, 579, 584, 621, 623 | 120, 155, 179, 231, 250, 259, 260, 269, 304, 333, 346 | 52, 54, 60, 62, 71 |
| HPV70\|Ref\|A | PPYKFHEHNNQ | LETP | APVQQRTEDRDEPRKD | VEQAVVFCNTR | SDESA |
| HPV70\|Qv27542\|A | L........D. | .... | ................ | .........N. | ..... |
| HPV70\|Qv27211\|A | .S.......D. | .... | .....K.......... | .........N. | ..... |
| HPV70\|Qv28219\|A | .........D. | .... | G..K.KN......... | ...T.....N. | .G... |
| HPV70\|Qv5102\|B | H.F.C...DDR | VDR. | ..LK.K........R. | DQH...SFHNK | FG.PS |
| HPV70\|Qv33565\|B | H....Y..DDR | VDR. | .L.K..........R. | .QH..LSFHNK | FGDPS |
| HPV70\|QV35039\|B | L....Y..DDR | VDR. | ...K.K.....KS.R. | .QH..LSFHNK | FGDPS |
| HPV70\|Qv27399\|B | .S......DDR | VDRL | ..LKLK.Q...K.K.. | .QH.A.SFHNK | LG.PS |
| HPV70\|Qv17574\|B | ...T.YQYDDR | VDR. | ..LK....NKNK.K.N | ..H.A.SFHNK | LG.PS |

| | E5 | NCR2 | L2 | L1 | URR |
|---|---|---|---|---|---|
| Position (nt) | 3928, 4070, 4074, 4083, 4092, 4117, 4138, 4142 | 4154, 4157, 4176, 4201 | 4225, 4264, 4343, 4385, 4496, 4514, 4571, 4583, 4670, 4688, 4727, 4732, 4760, 4772, 4904, 4917, 4949, 4964, 5030, 5076, 5114, 5159, 5177, 5183, 5207, 5278, 5306, 5307, 5507, 5553, 5558 | 5899, 5937, 5985, 6005, 6048, 6099, 6118, 6123, 6201, 6348, 6357, 6435, 6483, 6633, 6717, 6741, 6803, 6876, 6884, 6894, 7047, 7056, 7068, 7101 | 7122, 7131, 7132, 7150, 7156, 7231, 7288, 7291, 7336, 7458, 7459, 7460, 7462, 7482, 7494, 7496, 7501, 7506, 7521, 7522, 7540, 7573, 7596, 7607, 7628, 7642, 7724, 7748, 7759, 7773, 7839, 7863, 56 |
| HPV70\|Ref\|A | CGAACTAT | GG-C | CAACATTACTAATATATCACGTTCGAAGCCG | GCTGATT-TGGGCGATAAAAGAAG | -ATGTTTGGTTGAACGCAGATCCAGACATGTGG |
| HPV70\|Qv27542\|A | T....... | ..-. | ...T.....C.T.....TG.......G.... | .......-.......A...C.... | -TA.....A........C......C........ |
| HPV70\|Qv27211\|A | ........ | ..-. | .G.......C.......TG........A... | .......-....G..A........ | -TA.....A.......................A |
| HPV70\|Qv28219\|A | ..G....C | ..-T | ......C..C.......TG..AC..C..T.. | C....A.2.......A........ | -TA.....AC......T.A.........CT... |
| HPV70\|Qv5102\|B | .AG..GCC | AA1. | T.GA.CCCT.T.GTG.CTGAAA.TA...TTA | .TAAGA.2AAATTA.ACGG.A... | 3TAC.CG.ACCT..TT...CAGT..TA..T... |
| HPV70\|Qv33565\|B | .AGC..CC | AA1. | ..GA..CCT.T.GTG.CTGAAA..A...TTA | .TAAGAG2AAATTA.ACGG.A.G. | 3TACCCG.ACCT..TT...C.GTC.TAT.T.A. |
| HPV70\|QV35039\|B | .AG...CC | A.1. | ..GA.CCCT.T.GTGCCTGAAA..A...TTA | .TAAGA.2AAATTA.ACGG.AG.. | 3TAC.CGAACCTGCTT...C.GT..TAT.T.A. |
| HPV70\|Qv27399\|B | .AG.G.CC | AA1. | ..GAGCCCT.T.GTGCCTGAAA..A...TTA | .TAAGA.2AAATTA.ACGG.A..A | 3TAC.CG.ACCTGCTT...C.GT..TA..T.A. |
| HPV70\|Qv17574\|B | .AG...CC | AA1. | ..GA.CCCT.T.GTGCCTGAAA..A...TTA | .TAAGA.2AAATTAGACGG.A... | 3TAC.CG.ACCTGCTT...C.GT..TA..TGA. |

| | E5 | L2 | L1 |
|---|---|---|---|
| Position (aa) | 7, 56, 59, 62, 70, 77 | 6, 19, 175, 232, 237, 357, 367 | 104, 139, 177, 284, 435, 437 |
| HPV70\|Ref\|A | SMILFY | AKEHNNG | GSSETE |
| HPV70\|Qv27542\|A | L..... | ..V.... | .....D |
| HPV70\|Qv27211\|A | ...... | .R....S | ...... |
| HPV70\|Qv28219\|A | .V.... | .....T. | R..... |
| HPV70\|Qv5102\|B | .V..CS | V..Q... | .N.DA. |
| HPV70\|Qv33565\|B | .VL..S | ...Q... | .NADA. |
| HPV70\|QV35039\|B | .V...S | ...QH.. | .N.DA. |
| HPV70\|Qv27399\|B | .V.V.S | ...QH.. | .N.DA. |
| HPV70\|Qv17574\|B | .V...S | ...QH.. | .N.DA. |

1. insertion of 7bp (CTATTAC) between nt 4176 and 4177 within the NCR2 region;
2. insertion of 6bp (ACTGTA) between nt 6123 and 6124 within the L1 ORF;
3. insertion of 4bp (TTGC) between nt 7122 and 7123 within the URR region.

Fig. S1F
