# Supplementary figures and images for: Evolution and Taxonomic Classification of Alphapapillomavirus 7 Complete Genomes: HPV18, HPV39, HPV45, HPV59, HPV68 and HPV70
Source: PLoS One. 2013 Aug 16;8(8):e72565. doi: 10.1371/journal.pone.0072565 (PMC3745470; doi:10.1371/journal.pone.0072565)

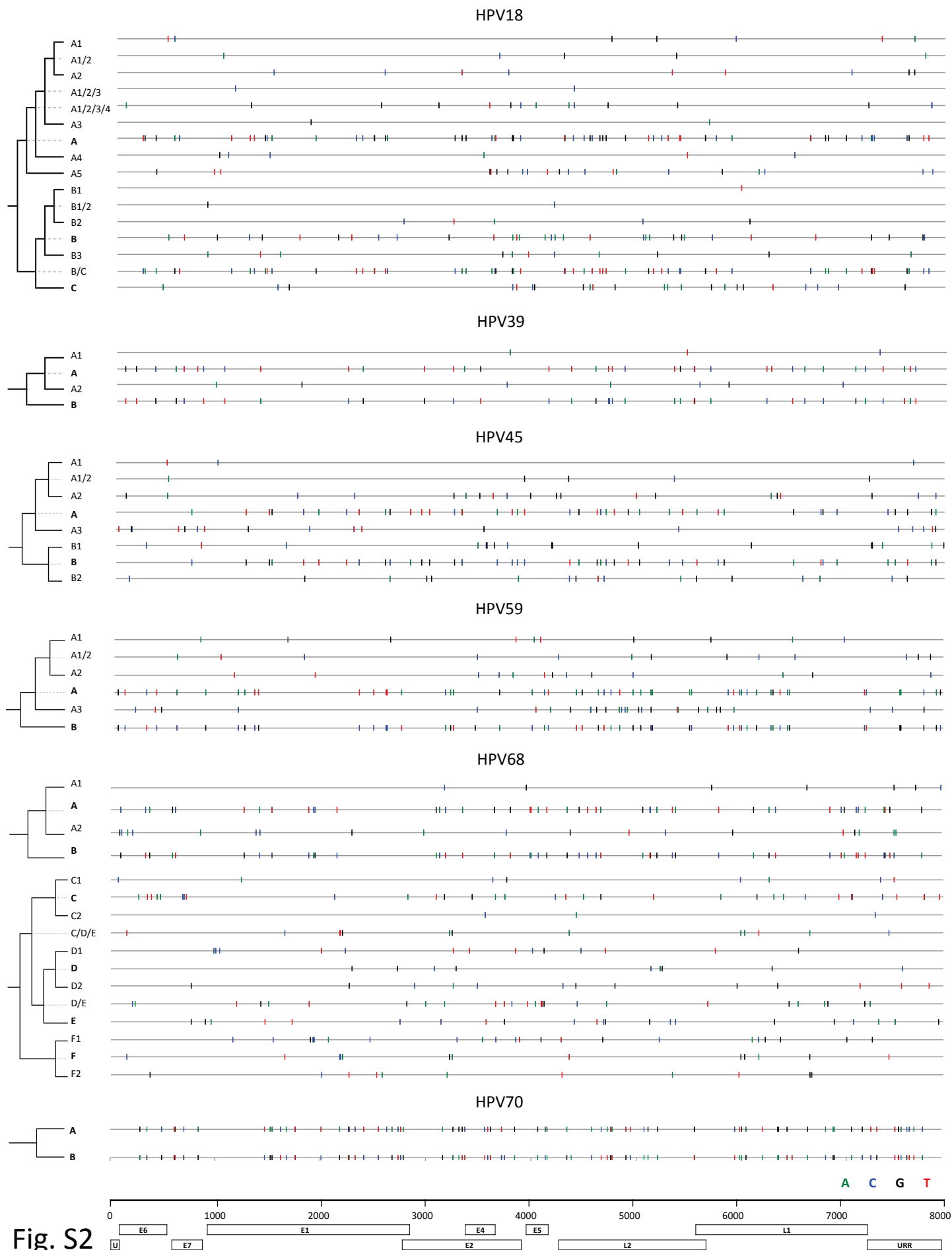

Fig. S2

Supplement: Figure S2 — Diagnostic lineage- and sublineage-specific single nucleotide polymorphisms (SNPs) of alpha-7 types. Lineage-specific SNPs were determined from alignments of type specific variants using the program MacClade. The location of variants across HPV lineage(s) and sublineage(s) are displayed to the right of the name of the clade from which the data were generated, as depicted in the phylogenetic trees in Figures 2-7. Numbered positions of SNPs in the genomes are shown in Figure S1. Regions of the genome are displayed below the x-axis for reference. The graphic output was generated using Microsoft Excel. (PDF) [file pone.0072565.s005.pdf]

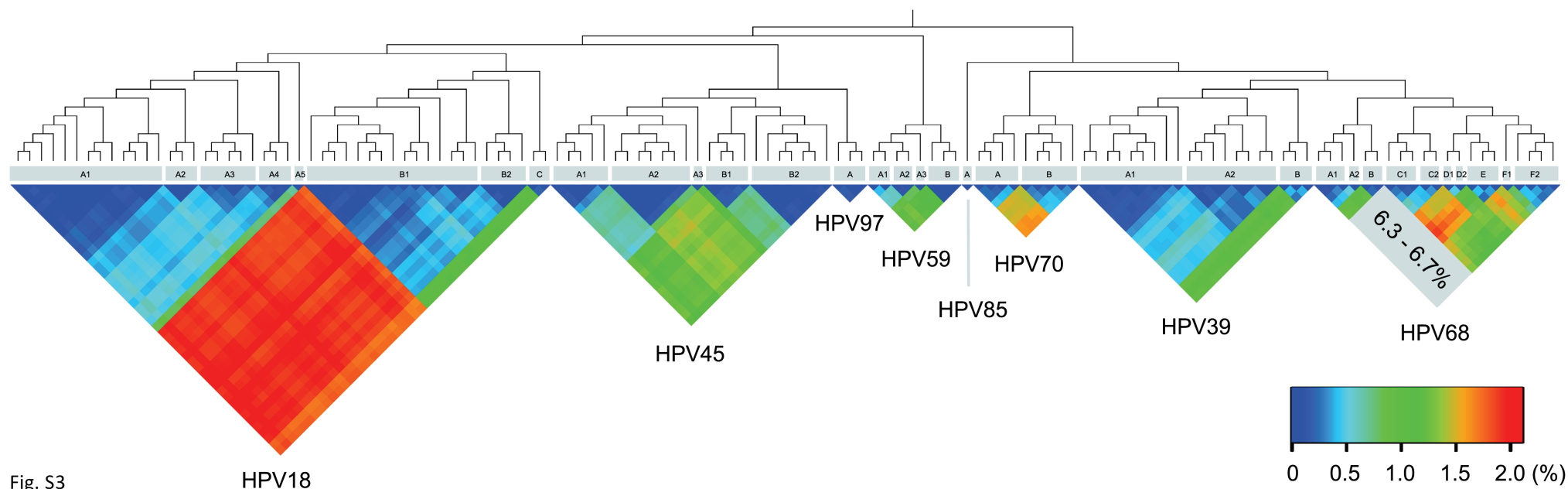

Supplement: Figure S3 — Heatmap display of alpha-7 HPV variant genome nucleotide sequence differences. P-differences calculated based on the complete genome nucleotide sequence alignment of each type were measured and represented as a heatmap, scaled such that complete identity (0.0% difference) is displayed as blue and the maximum difference (2.1%) as red. A phylogenetic tree indicating the position and name of each lineage and sublineage is shown above the heatmap. (PDF) [file pone.0072565.s006.pdf]

A.

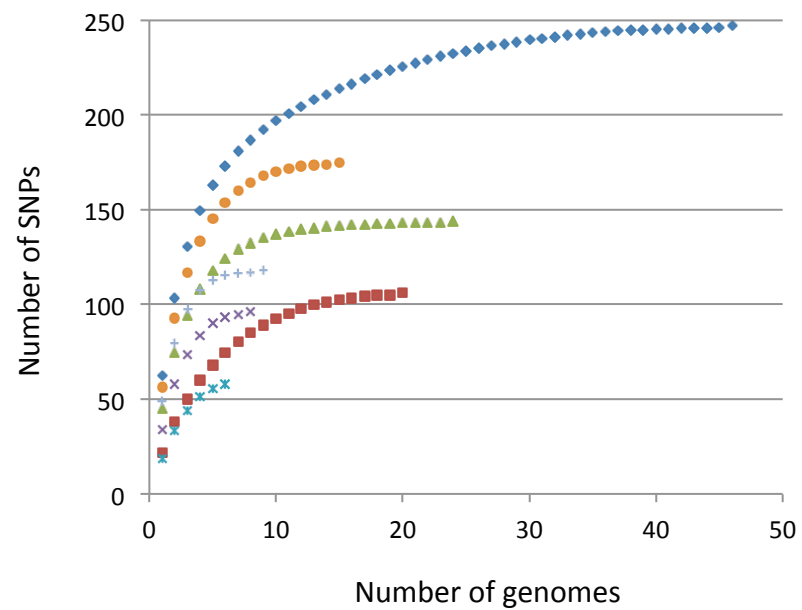

B.

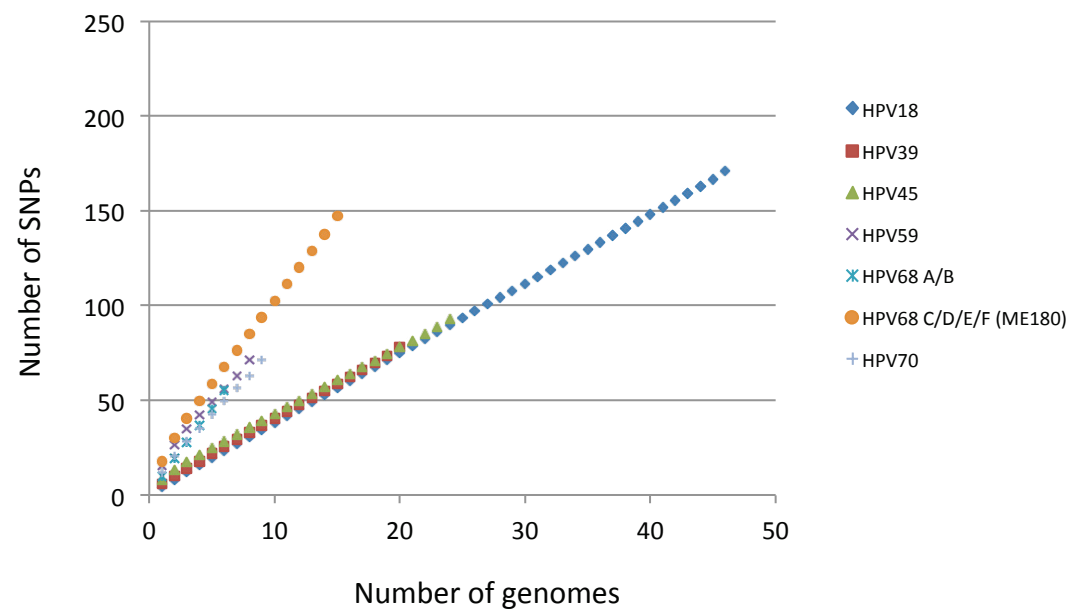

Fig. S4

Supplement: Figure S4 — Rarefaction curves of alpha-7 HPV parsim-informative (A) and singleton (B) SNPs. The program EstimateS was used to illustrate the curves. The Y-axis represents the total number of SNPs (indels were counted as one event equal to a single SNP). The X-axis shows the number of sequenced isolates. The curve generated for variants of each HPV type are displayed by different lines as indicated by the key to the right of the curves. HPV68 variants were split into two groups, HPV68A/B and HPV68C/D/E/F (HPV68-ME180). For reference, the number of variable nucleotide positions for HPV18, HPV39, HPV45, HPV59, HPV68 and HPV70 genomes are 5.4%, 2.4%, 3.0%, 2.1%, 9.8% (1.5% of HPV68A/B, and 4.2% of HPV68C/D/E/F) and 2.4%, respectively (Table 1). (PDF) [file pone.0072565.s007.pdf]
