# Supplementary material for: Evolution and Taxonomic Classification of Alphapapillomavirus 7 Complete Genomes: HPV18, HPV39, HPV45, HPV59, HPV68 and HPV70
Source: PLoS One. 2013 Aug 16;8(8):e72565. doi: 10.1371/journal.pone.0072565 (PMC3745470; doi:10.1371/journal.pone.0072565)
Supplement: Figure S5 — Representation of an alpha-7 HPV genome and type-specific ORF/region sizes. Each region or ORF of the HPV genome is indicated outside the double-stranded circle. Lengths of each ORF and region are indicated by the histogram pointing to the region/ORF in the figure. The length in nucleotide sequences (bp) for each alpha-7 HPV genome is indicated with the minimal and maximal lengths represented by the bars with dots or highlighted in grey, respectively. The diagram of the HPV genome is not drawn to scale and the histogram for each ORF/region is presented in a different range of values. (PDF) [file pone.0072565.s008.pdf]

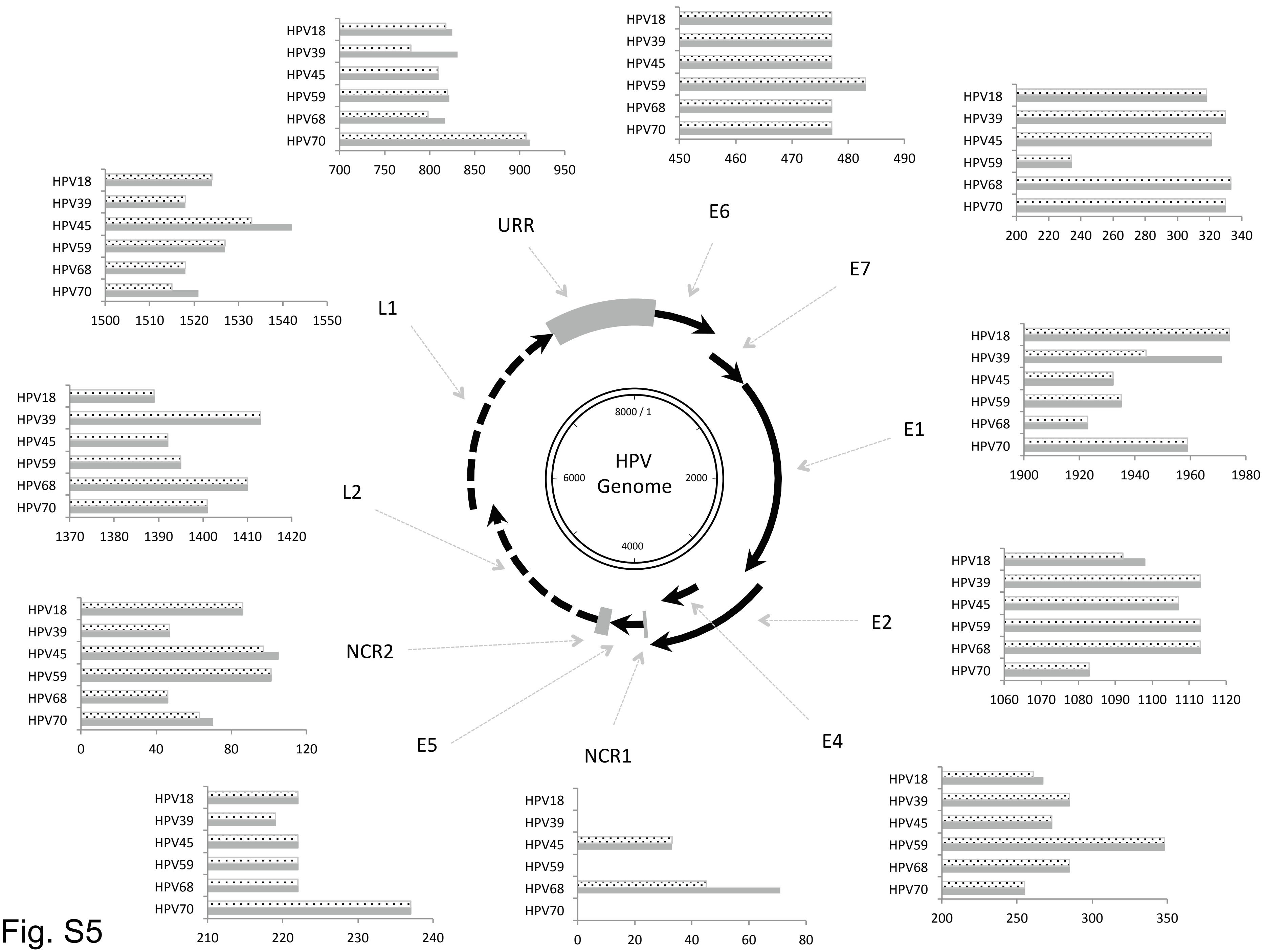
HPV Genome
8000 / 1
2000
4000
6000
URR
E6
E7
E1
E2
E4
NCR1
E5
NCR2
L2
L1
HPV18
HPV39
HPV45
HPV59
HPV68
HPV70

Fig. S5
